# Isoflavones

**DOI:** 10.3390/molecules24061076

**Published:** 2019-03-19

**Authors:** Ludmila Křížová, Kateřina Dadáková, Jitka Kašparovská, Tomáš Kašparovský

**Affiliations:** Department of Biochemistry, Faculty of Science, Masaryk University, Kotlarska 2, 61137 Brno, Czech Republic; Ludmila.S@seznam.cz (L.K.); 147047@mail.muni.cz (K.D.); 20829@muni.cz (J.K.)

**Keywords:** isoflavones, phytoestrogens, daidzein, genistein, glycitein, formononetin, biochanin A, equol

## Abstract

Phytoestrogens are naturally occurring nonsteroidal phenolic plant compounds that, due to their molecular structure and size, resemble vertebrate steroids estrogens. This review is focused on plant flavonoids isoflavones, which are ranked among the most estrogenic compounds. The main dietary sources of isoflavones for humans are soybean and soybean products, which contain mainly daidzein and genistein. When they are consumed, they exert estrogenic and/or antiestrogenic effects. Isoflavones are considered chemoprotective and can be used as an alternative therapy for a wide range of hormonal disorders, including several cancer types, namely breast cancer and prostate cancer, cardiovascular diseases, osteoporosis, or menopausal symptoms. On the other hand, isoflavones may also be considered endocrine disruptors with possible negative influences on the state of health in a certain part of the population or on the environment. This review deals with isoflavone classification, structure, and occurrence, with their metabolism, biological, and health effects in humans and animals, and with their utilization and potential risks.

## 1. History

The word “phytoestrogen” comes from the Greek term for plant (“*phyto-*”) and from the term “*estrogen*”, that is a hormone that influences the female fertility in vertebrates. Phytoestrogens are compounds found in plants that, due to their molecular structure and size, resemble estrogens, in particular to estradiol (17-β-estradiol, E2), and that exert estrogenic and/or antiestrogenic effects [1]. Their occurrence was first reported in the 1940s in connection with the “clover disease” [2], which appeared in Australia in sheep grazing subterranean clover (*Trifolium subterraneum*). The “disease” manifested the symptoms of diverse reproduction disorders. Young immature animals showed signs of estrus, ewes were not able to get pregnant, and those that were pregnant often aborted. Increased incidence of uterine abnormality and endometriosis was reported [2]. Abnormal development of the mammary gland or abnormal lactation, uterine prolapse, or uterine dystocia as a result of incomplete cervical dilation are also ranked among the symptoms [3]. Lower sperm count and motility was reported in rams [4]. The main plant-derived constituent responsible for the clover disease formation was determined to be formononetin [5]. Following intraruminal administration of formononetin, equol is excreted in the sheep urine [6]. High levels of circulating equol were found in the sheep suffering from the clover disease as a result of grazing several indigenous clover species that contained high formononetin concentrations [6]. Equol was found even in the urinary calculi of cattle and sheep [7].

Equol was first isolated from the urine of pregnant mares in 1932, and it is the source material (equine urine) that gave this compound its name [8]. Substantial equol amount was soon found also in the urine of stallions and non-pregnant mares. That way, the original hypothesis of the connection between equol and high estrogen concentration in the pregnant organism was disproved. Afterwards, seasonality of equol occurrence in the equine urine was discovered, as during autumn, the equol content declined and, in winter, equol could not be detected at all in the urine [9]. Thanks to the interconnection of the information and knowledge available at that time, the dietary source of equol was discovered, and it was given the main role in causing the disorders of estrogen balance in sheep [10,11]. Since then, equol occurrence in the urine or plasma was reported in many animal species including pigs [12], cattle [13], poultry [14], primates [15], laboratory rodents [16], and dogs [17]. Fifty years later, equol was also identified in human blood as a metabolite of the soybean isoflavones daidzin and daidzein [18].

## 2. Isoflavone Classification and Chemical Structure

Phytoestrogens are naturally occurring nonsteroidal phenolic plant compounds and can be divided into two main groups: flavonoids and non-flavonoids [19]. Flavonoids include isoflavones, coumestans and prenylflavonoids, and non-flavonoids include lignans.

Genistein (7,4′-dihydroxy-6-methoxyisoflavone), daidzein (7,4′-dihydroxyisoflavone), glycitein (7,4′-dihydroxy-6-methoxyisoflavone), biochanin A (5,7-dihydroxy-4′-methoxyisoflavone), and formononetin (7-hydroxy-4´-methoxyisoflavone) belong to isoflavone phytoestrogens. Equol, as a daidzein metabolite, is sometimes also ranked among this group [19], but as Setchell et al. [20] point out, equol is not a phytoestrogen, as it is not a natural plant compound. It is solely a metabolic product of intestinal bacteria. Isoflavones are ranked among the most estrogenic compounds. The main source of isoflavones are legumes from the family *Fabaceae* [21], namely soybean (*Glycine max*) as a source of daidzein, genistein, and glycitein and red clover (*Trifolium pratense)* as a source of formononetin and biochanin A.

Flavanone liquiritigenin (7,4′-dihydroxyflavanone) is the precursor of daidzein, formononetin, and glycitein; the precursor of genistein and biochanin A is naringenin (5,7,4′-dihydroxyflavanone) [22]. Isoflavones may occur as aglycons or as glycosides (Table 1). They may form 7-*O*- or, in some plant species, 8-*C*-β-d-glycosides, 6″-*O*-malonylglycosides, or 6″-*O*-acetylglycosides [23].

## 3. Isoflavone Role in Plants

In plants, phytoestrogens do not function as hormones, but as phytoalexins, i.e., low-molecular compounds synthesized and accumulated in plants during stress and microbe attacks. These active defense compounds have fungistatic, antibacterial, antiviral, and antioxidant properties [26]. They also prevent angiogenesis, thereby being important in the fight against malignant tumors [27].

Isoflavonoids play many roles in plant-microbe interactions, including rhizobia-legume symbiosis and defense responses [28]. Isoflavones are essential for nodulation because of their ability to induce the nodulation genes [29]. Further, isoflavones often serve as precursors of more antimicrobial isoflavonoid phytoalexins. Isoflavonoid phytoalexins include isoflavones, isoflavanones, pterocarpans, isoflavans, and coumestans [26]. Often, more than one type can be found in a single plant species and the same isoflavonoids may be produced by different plant species. In soybean, daidzein is metabolized to produce glyceollins, which serve as defense mechanisms against several, primarily fungal, pathogens [30]. In the model legume *Medicago truncatula*, formononetin is metabolized into medicarpin, a phytoalexin that is conncected with pathogen resistance [31]. Other isoflavonoid phytoalexins include, e.g., formononetin derivative pisatin from pea (*Pisum sativum*) or genistein derivative kievitone and daidzein derivative phaseollin from french bean (*Phaseolus vulgaris*) [26].

The amounts of phytoestrogens produced by a plant depend mainly on growing conditions and on the plant cultivar. The isoflavone concentration raises sharply during stress (e.g., lowered humidity, pathogen attack, or plant diseases) and is, to a large extent, influenced by environmental and climatic conditions like temperature, precipitation, harvest period, or soil fertility. The final isoflavone concentration is also influenced by the post-harvest processing [25,32,33].

## 4. Isoflavone Occurrence Relevant for Animals and People

Isoflavones occur primarily in legumes [23]. In the diet of farm animals, soybean (*Glycine max*), red clover (*Trifolium pratense*) and white clover (*Trifolium repens*), and alfalfa (*Medicago sativa*) are important isoflavone sources. In soybean, isoflavone content (mainly daidzein, genistein, and their conjugates) amounts to 1.2–4.2 mg/g dry weight [1]. Red clover contains the most phytoestrogens, 10–25 mg/g dry weight, whereas white clover contains 0.5–0.6 mg/g dry weight [34]. These phytoestrogens comprise mostly isoflavones, mainly formononetin, which can account for 0.8–11 mg/g dry weight [35]. In alfalfa, the isoflavone content amounts only to 0.05–0.3 mg/g dry weight [36]. The content of isoflavones depends on the plant part, growth stage, cultivar, growing conditions, and preservation method [25,33].

In connection with the human diet, the main sources of isoflavones are soy and soy-derived products. The content of isoflavones in soy beans is approximately 1.5 mg/g, whereas the contents in soy-derived foods are usually lower [37]. Other dietary sources of isoflavones include chickpeas and beans, and small amounts of isoflavones are also contained in other plant products, such as fruits, vegetables, and nuts [38]. Apart from this, in the western (primarily American) population, the consumption of cow’s milk and dairy products was found to participate in the total isoflavone intake [39]. As isoflavone sources, red clover products are used also in human dietary supplement production for the reduction of menopausal symptoms in women [40].

## 5. Isoflavone Metabolism in Animals

Phytoestrogen metabolism was described in many animal species including sheep [12], cattle [13], goats [41], or poultry [14]. Soybean or red clover, which contain high isoflavone concentrations, are commonly used in the diet of ruminants, namely sheep and dairy cows, and the proportion of this feed in the feeding ration of these farm animals is relatively high. That is why the research focuses on the possible impact of dietary isoflavones on the reproductive performance of ruminants and also on the possibility of functional food production.

The raw materials used for the feed production contain isoflavone glycosides, which are not active as estrogens [42] and are hydrolyzed to aglycons by ruminal microorganisms [43,44]. Many studies have proved that animals, unlike humans, commonly produce equol [12,15,16,17]. In ruminants, soybean isoflavones (daidzein, genistein, glycitein) and red clover isoflavones (formononetin, biochanin A, daidzein, genistein) are metabolized to equol, to metabolically inactive *p*-ethyl-phenol [45], or marginally to *O*-desmethylangolensin by rumen microbiota. The biological activity of the latter metabolite is similar or weaker than that of equol [46]. Biochanin A is demethylated to genistein and is further converted to the ring cleavage metabolites *p*-ethyl-phenol and organic acids [11,43]. These metabolites have no estrogenic effect. Formononetin is primarily demethylated to daidzein [43] and further hydrogenated and cleaved to form equol [46]. The in vitro studies that described the incubation of formononetin and biochanin A in the bovine rumen fluid showed that the half-lives were: 4.3 h for formononetin, 9.3 h for daidzein, 3.9 h for biochanin A, and 5.5 h for genistein [44].

The metabolic conversion of isoflavones takes place mainly in the rumen. Njåstad et al. [47] found out that only 0–9% of ingested biochanin A and genistein and 7–16% of formononetin and daidzein were found in the omasum of dairy cows. Similar results were described in sheep. In the abomasum, neither biochanin A nor genistein were found and 12% of the daily ingested amount of formononetin and daidzein [11]. Furthermore, the percentage of isoflavones present in the omasum decreased with increasing isoflavone intake [47]. This is caused by the microbiota adaptation, which takes 6–10 days and makes the metabolism more efficient [48]. Furthermore, other factors, like the nutrient contents, are involved in isoflavone metabolism. Generally, higher equol production is connected to hay diets than to concentrate-rich diets [49]. Equol was the dominant isoflavone found in the digesta of both cattle [47] and sheep [11]. In the latter study [11], equol was found in the liquid part of the digesta, whereas in the first study [47], equol was surprisingly found attached to the large particles. This could be explained by the hypothesis that the main part of equol dissolved in the fluid was already absorbed in the rumen, and only the part that was located inside or close to the rumen microorganisms attached to the feed particles passed to the omasum [47].

Isoflavone aglycons are absorbed not only in the rumen [47], but also in the gut [50]. The isoflavone metabolism in the intestine of ruminants was not yet thoroughly described, but it may be assumed that the metabolic conversions are similar to those found in humans (glucuronidation, sulphatation, enterohepatic circulation). Compared to sheep, Lundh et al. [50] found a much lower conjugation capacity in the epithelial tissue of the bovine gastrointestinal tract. This may be the reason why less equol is absorbed in the bovine rumen and more equol passes through the bovine gastrointestinal tract, as compared to sheep.

In ruminants, equol and daidzein may be distributed into diverse tissues, but the distribution is not uniform [51]. Isoflavone levels in the kidneys or in the liver may be high, but isoflavone penetration into the brain tissue is minimal. The reproductive organs contain higher isoflavone levels then the heart, muscles, or lungs. Isoflavones are distributed into the tissues as glucuronides. They are likely not accumulated in the tissues, except for the kidneys [51].

Some older studies indicated that in the sheep fed with red clover, 16 times more equol is excreted in the urine then in the feces [11]. In contrast, Tucker et al. [52] or Njåstad et al. [47] assume from the results of experiments with heifers and dairy cows that the main equol excretion route is via feces. These results indicate that there are substantial differences in the isoflavone metabolism between sheep and cattle.

Relatively large quantities of isoflavones are excreted in milk, the predominant isoflavone being equol [3,35,53]. The red clover isoflavone transfer to milk is well described. When feeding red clover, fresh or preserved (silage), the level of equol in milk may range between 15 and 650 µg/L [35,54,55]; some studies, however, show levels reaching 1000–1500 µg/L [56], or even higher (1700 µg/L) [57]. When feeding white clover, the level of equol in milk is about four times lower, compared to red clover [35]. A larger equol quantity was described in organic milk than in conventionally produced milk [33]. When feeding soybean components, equol reaches a much lower level, namely 14–186 μg/L, in milk [53,58,59,60,61]. The above-mentioned levels are proportional to the amounts of soybean components in the dairy diets.

The quantitative aspect of isoflavone transfer from feed into bovine milk has not been thoroughly described so far. Accessible are mainly the data that concern clovers. Mustonen et al. [54] realized a strong dependence of equol concentration in the plasma on formononetin intake (y = 0.071x + 2.75 R^2^ = 0.71) and by contrast, only a weak dependence of equol concentration in milk on formononetin intake (y = 0.0035x + 0.358 R^2^ = 0.20). The formononetin and daidzein carry-over rates from feed to milk are 0.92–1.2 for white clover, 0.21–0.24 for red clover [35], and 1.23 for grass/clover silage [62]. In the case of soybean isoflavones, the daidzein carry-over rate ranges from 0.50 [61] to 1.3 [53]. Flachowsky et al. [58] detected a higher value (2.0) and realized that the total isoflavone transfer to milk decreased with increasing isoflavone intake according to the relation y = −0.0001x + 0.0006 (R^2^ = 0.69). This finding is in accordance with Steinshamn et al. [35], Mustonen et al. [54], Andersen et al. [62], Höjer et al. [56], Třináctý et al. [53], or Njåstad et al. [47]. All these authors report a higher isoflavone carry-over rate to milk associated with a lower isoflavone intake. In an effort to explain these results, Steinshamn et al. [35] assume that conjugation in the gastrointestinal tract and reconjugation in the liver prior to the transport and secretion in the mammary gland may be limiting. Furthermore, compliant with the opinion of Turner [63], they believe that the permeability of the mammary gland epithelial cells for estrogenic compounds may be limited. Njåstad et al. [47] also conclude that the limiting factors are reconjugation and absorption across the rumen and intestinal wall. These suggestions are supported by the fact that both conjugation reactions and active transport of substrates from blood to the mammary gland tissue usually follow the Michaelis-Menten saturation kinetics [64].

Schwen et al. [65] studied the metabolism of S-equol in rats and monkeys. In the rat plasma, they detected conjugated 4′-glucuronide, conjugated 7-sulphate, and diconjugated 7-sulphate-4′-glucuronide as main metabolites. In monkeys, the metabolism was intensive as well, and in the plasma, 4′-glucuronide and 7-sulphate-4′-glucuronide were found. The urine (in both rats and monkeys) contained primarily 4′-glucuronide. The pathways typically used to metabolize isoflavones are less efficient in carnivores. In cheetahs, 5–15% of daidzein and genistein is excreted in the urine and 40–70% is excreted in the feces [66]. In the urine, isoflavones are present mainly as conjugates (glucuronides and sulphates) whereas, in the feces, the main part of isoflavones remains unconjugated. Equol was not detected in the samples of cheetah plasma, urine, and feces [66].

## 6. Biological and Health Effects of Isoflavones in Animals

### 6.1. Estrogen Activity

Apart from the above-mentioned clover disease in sheep [2], which led to the discovery of the phytoestrogens, many observations of other animal species proved the negative influence of soybean isoflavones on the female reproductive ability.

#### 6.1.1. Sheep

Formononetin and daidzein contained in clover used for sheep grazing is metabolized in the sheep rumen into equol (see Section 6) [12], which was determined as the main cause of reproduction disorders in sheep [10,11]. The isoflavone pathophysiological effect on the sheep reproduction was manifested, when equol plasma concentrations were approaching 20 µmol/L [10]. The pasture vegetation estrogenicity depends on clover proportion. The vegetation that contains up to 0.3% of formononetin in the dry matter does not cause reproduction disorders in sheep, while grazing of the vegetation containing more than 0.8% of formononetin in the dry matter may lead to reproduction difficulties [67]. A lower isoflavone intake causes temporary infertility, a long-term exposure may lead to permanent infertility [48,67].

The research in the field of the isoflavone influence on sheep reproduction carried out recently is ambiguous. Sakakibara et al. [68] state that high formononetin concentrations increased the number of ewe miscarriages and stillborn lambs. Therefore, they do not recommend the feeding of isoflavone-rich diets during the breeding season and early pregnancy. Mustonen et al. [3] observed the effect of the red clover silage feeding on reproduction indicators of sheep over five months. Even though the authors did not notice any fertility reduction (the same litter size), they determined that, in pregnant animals, the total uterine weight (including the content) was higher in the sheep fed with the clover silage. This was namely because of a higher fetal fluid amount, which may increase the risk of vaginal prolapse before the birth. The progesterone concentration in the blood serum was lower in animals fed with the clover silage than in the control group throughout the experiment.

#### 6.1.2. Cattle

Recently, several in vitro and in vivo studies were conducted on the effect of soybean isoflavones on cattle reproductive organs, estrous cycle, and hormonal profile [69,70,71,72,73].

Isoflavones have been found to act as antagonists or as agonists in relation to endogen estrogens [1]. In cows, endogen estrogens regulate the estrous cycle through influencing of the prostaglandin synthesis [74]. The prostaglandin F2α (PGF2α) has a luteolytic effect, whereas the prostaglandin E2 (PGE2) has a luteoprotective effect [75]. A mutual ratio of PGE2 and PGF2α is of principal importance for correct development, function, and maintaining of the corpus luteum (CL), pregnancy identification, uterus mucosa readiness for the nidation of the fertilized ovum, the nidation itself, and embryonic development [76]. Although soy isoflavones have been proved to influence the synthesis of both PGF2α and PGE2 [69], Woclawek-Potocka et al. [77] found out that soybean isoflavones and their metabolites stimulate preferentially the synthesis of PGF2α during the luteal phase of the estrous cycle. The stimulation of the PGF2α production compared to the PGE2 production causes disruption of the optimal ratio between these prostaglandins. This may be one of the reasons for early embryonic mortality or later abortions in pregnant animals [69,76]. In non-pregnant animals, the PGF2α stimulation during the estrous cycle (during the late luteal and follicular phase of the cycle) may have a positive effect on the mechanisms responsible for luteolysis and ovulation commencement [77]. The negative effects were manifested namely in the heifers and in the cows on their first lactation. In older dairy cows, whose feeding ration contained a similar soybean amount, only a tendency to higher PGF2α levels (*p* = 0.095) was observed [73]. Neither the progesterone concentration in the blood plasma nor the length of the estrous cycle were influenced by isoflavone intake of up to 3 g/d [69,73].

High concentrations of active isoflavone metabolites were found after the soybean feeding in the CL tissue of heifers [72], at the same time a lower progesterone concentration was found compared to the control group. The isoflavone presence in CL may be assumed to directly disrupt its function by inhibiting the progesterone secretion. This may cause various disorders during the early pregnancy, including early embryonic death [78]. However, the progesterone production is stimulated also by other mechanisms like luteinizing hormone (LH), luteal, and/or ovarian PGE2 [72].

Woclawek-Potocka et al. [69,70,77] studied the local isoflavone effects on the secretion function of cattle endometrium in many in vitro experiments. They determined that isoflavone metabolites are much more effective disruptors than the parent isoflavones because they possess a greater affinity to estrogen receptors. Further, they found lower daidzein and genistein concentrations in the plasma of heifers in the beginning and in the end of pregnancy, as compared to control animals in the middle of luteal phase of the estrous cycle [71]. In the plasma of heifers in the beginning of pregnancy, they discovered a decrease in the isoflavone concentration, which started 3 h after feeding. The authors assume that this metabolism acceleration, which leads to increased equol and p-ethyl phenol concentrations, was caused by β-glucuronidase activation, occurring not only in the early pregnancy but also during the mobilization of the immune system [79]. In late-pregnant heifers, this phenomenon was not noticed. Therefore, Woclawek-Potocka et al. [45] assumed that there was a hormonal mechanism that slows down isoflavone metabolism and decreases their absorption.

### 6.2. Isoflavone Effect on Health and Productivity of Farm Animals

According to Mohanty et al. [80], young plants with high phytoestrogen activity possess galactopoietic properties. This is in accordance with Dewhurst et al. [81], or Vanhatalo et al. [82], who found a higher dry matter intake and a higher milk production with a more favorable fatty acid profile (higher polyunsaturated fatty acid proportion) in dairy cows fed on red clover silage as compared to dairy cows fed on grass silage. Liu et al. [83] noticed a higher milk production with higher fat and protein contents following daidzein administration in late-lactating dairy cows. Following the addition of 300 and 400 mg of daidzein into the feeding ration of lactating dairy cows exposed to heat stress, higher immunoglobulin G, interferon-α, and interleukin 2 levels were detected in their blood serum, while total protein and albumin levels did not change. This reflects an increase of the immune functions and heat stress resistance of the dairy cows [83].

Moorby et al. [84] determined that, at the same daily dry-matter intake, lambs grazing red clover with high formononetin concentration reached higher weight gains than lambs grazing red clover with a lower formononetin content or perennial rye-grass. Similar results are reported by Speijers et al. [85].

Daidzein inclusion into the feeding ration of laying hens improved the pre-ovulation follicular development [86], increased the egg weight and the laying performance [87], increased the eggshell thickness and strength and raised the level of calcium in the shell [88]. Additionally, daidzein supplementation positively influenced the diversity of ileal microbiota [87].

## 7. Isoflavone Metabolism in Humans

The main dietary source of isoflavones in humans are soybean and soybean products, which contain mainly daidzein and genistein [23]. Phytoestrogen dietary supplements made from red clover extracts, which are becoming more and more popular as an alternative therapy for the treatment of menopausal symptoms, indirectly provide a source of daidzein, as the methoxylated isoflavone formononetin from red clover is effectively transformed into daidzein in the human gastrointestinal tract [89].

The metabolism of soybean isoflavones in humans is well described in the literature [90]. Aglycons are absorbed in the proximal part of the small intestine by passive diffusion and they reach maximal blood concentration during one hour after their infusion into the duodenum [91]. After oral ingestion, the peak plasma isoflavone concentrations are reached after 7.2 to 7.4 h and their concentrations in plasma depend on the oral dose [92]. One hour after aglycone intake, the composition of genistein and its glucuronide metabolite in peripheral blood is dose-dependent containing 50–100% of the glucuronide metabolite [91]. In the soybean protein and in most soybean products, however, the isoflavones are conjugated to sugars. Unlike the aglycons, β-glycosides cannot be absorbed due to their higher hydrophilicity and higher molecular mass [93]. They become bioavailable and can be metabolized only when hydrolyzed [20], malonylglycosides being less bioavailable then β-d-glycosides [94].

Isoflavones can be hydrolyzed along the entire length of the gastrointestinal tract, but mostly, they are hydrolyzed in the jejunum [95] by the cooperation of the brush border membrane and bacterial β-glucosidases [96], which are active from relatively early life stages. Their action releases the aglycons, which are subsequently absorbed across the intestinal epithelium [97]. After the absorption, genistein and daidzein are metabolized by UDP-glucuronyl transferase to β-glucuronides, and to a lesser extent by sulphotransferases to sulphate esters in the intestinal mucosa cells [98]. The conjugation can happen in one or two (4′ or 7′) locations of the isoflavone ring and may also take place in the liver. These metabolites (mono- and diglucuronides, mono- and disulphates, and sulphoglucuronides of daidzein and genistein) can be found in the plasma [99], are excreted in the bile and deconjugated in the distal part of the intestine. This allows them to be absorbed again and be part of the enterohepatic circulation [100].

Part of the isoflavones passes to the large intestine, where the glycosylated, sulphated and glucuronidated forms are deconjugated by bacterial enzymes and subsequently absorbed or further metabolized by intestinal microflora [95,97,101]. Daidzein is metabolized to dihydrodaidzein, which is further converted to equol or *O*-desmethylangolensin (*O*-DMA) [102]. Equol occurs in two forms, S- and R-equol; however, human intestinal microflora synthesizes only S-equol [103,104]. Genistein is converted to dihydrogenistein and further metabolized to *p*-ethyl-phenol and 6-hydroxy-*O*-DMA. Glycitein is stable, because the immediate proximity of the 6-methoxyl and the 7-hydroxyl groups blocks the demethylation. Therefore, glycitein is not converted to daidzein and so it is not a precursor of equol [92,103].

Toro-Funes et al. [105] studied the isoflavone metabolism in different cell types. They determined that endothelial cells take up genistein and daidzein and metabolize them to methoxy-genistein-glucuronides, methoxy-genistein-sulphates, and methoxy-daidzein-glucuronides. Equol is also taken up by these cells, but it is not metabolized. On the contrary, in the liver cells and in the epithelial cells of the intestine, not only were glucuronide and sulphate conjugates of genistein and daidzein produced, but also sulphate conjugates of equol.

Equol is absorbed more efficiently across the large intestinal wall than daidzein [97]; this fact is evident when the plasma concentrations of these isoflavones are compared [95]. During the first 4 h, the plasma concentration of equol is inconsiderable. The plasma concentration reaches its maximum 24 h after the ingestion of isoflavones, then it gradually decreases, but remains elevated during the next 24 h [95]. The excretion of equol metabolites in the urine is variable. Equol and traces of its mono- and dimethoxylated conjugates may be found in the urine [106]. In healthy people that do not consume soybean, equol is normally not present in the urine. Its production depends solely on the intestinal microflora. Germ-free animals do not excrete equol [107], just as it cannot be found in the plasma of the infants fed with infant formulae [108,109]. After isoflavone consumption, equol and its metabolites are excreted only by some human individuals [1]. About 40–70% of the adult population do not excrete equol in the urine, even if they have consumed soybean products or pure isoflavones [89,92,110], because, for a reason that is not known, they do not harbor specific intestinal bacteria involved in the metabolism of daidzein to equol [111].

Therefore, the terms “equol producers” and “equol nonproducers” were defined so that the two abovementioned groups can be differentiated in the population. Equol nonproducers are defined as subjects, whose plasma concentration of equol is lower than 40 nmol/L (10 µg/L) under strictly-defined conditions, and equol producers are defined as subjects whose plasma concentration of equol is higher than 83 nmol/L (20 µg/L). These values were determined empirically [89]. This discrimination may also be made based on equol concentration in the urine: equol producers excrete more than 1000 nmol/L [110]. Currently, the long-term stability of the ability to metabolize daidzein to equol is being discussed. The ability to produce equol was considered relatively stable, because many studies indicated that the subjects are not able to change their equol-producer status [92]. However, recent studies showed that the ability to produce equol is only stable in the course of 1 year [112], 1–3 years [113], or 1–5 years in 85% of the subjects [114]. In a study with 350 postmenopausal women, Franke et al. [115] observed the changes of the equol-producer status in up to 35% of the women during 2.5 years. Franke et al. [116] presume that the differences between the studies may be caused by the method used to define equol producers (different limit equol concentrations may be seen e.g., in [117] vs. [118]) and by the matrices used (plasma vs. urine). Urine has been shown to be a better matrix for the equol-producer status classification, because it enables to monitor the time-course changes and is more accurate for the evaluation of isoflavone-exposure influence than blood, where equol is eliminated relatively quickly [115,116]. Using the ratio of equol concentration to daidzein concentration makes the equol-producer status classification substantially more precise [119]. Rapid and sensitive analytical methods are implemented [120] and further accuracy improvement and standardization of the methods for equol-producer status classification are expected. This will be reflected in the result interpretation of current and future studies.

The level of daidzein conversion to equol can be influenced by saccharide intake. Cassidy [121] and Setchell and Cassidy [101] proved this using in vitro methods and Lipovac et al. [122] using in vivo methods. The increase of non-starch polysaccharide intake, which stimulates bacterial fermentation, raises the production level of equol, and on the contrary, conditions that simulate a low saccharide intake, block equol production [122]. This fact indicates that other food components like fat [110], fiber, or higher proportion of plant proteins [123] may also influence isoflavone metabolism. The profile and diversity of intestinal microflora influence isoflavone metabolism substantially [124].

Concrete bacterial strains involved in the isoflavone metabolism are not known and these issues are currently being addressed. Elghali et al. [125] stated that *Bifidobacteria* sp. (*B. breve* and *B. longum*) were able to transform daidzein into equol. Shimada et al. [126] found out that the *Lactococcus* strain 20–92 participated in daidzein conversion to equol via dihydro- and tetrahydrodaidzein. The ability of the strain Julong 732 (*Eggerthella* sp.) to convert dihydrodaidzein to equol was reported [127]. Kim et al. [128] isolated bacteria that belong to *Lactococcus* sp. (MRG-IFC-1 and MRG-IFC-3) and *Enterococcus* sp. (MRG-IFC-2) from human fecal samples, which were able to hydrolyze the C-glycosides. Other bacteria involved in the isoflavone metabolism include *Escherichia coli* HGH21, *Clostridium* sp. HGH136 [93], a clostridium-like bacterium [129], *Eubacterium ramulus* [130], *Lactobacillus* sp. Niu-O16 [131], *Adlercreutzia equolifaciens*, *Slackia isoflavoniconvertens* [111], or mixed cultures like that of *Bacteroides ovatus*, *Streptococcus intermedius*, and *Ruminococcus productus* [132], or *Lactobacillus mucosae* EPI2, *Enterococcus faecium* EPI1, *Finegoldia magna* EPI3, and *Veillonella* sp. strain EP [97]

## 8. Biological and Health Effects of Isoflavones in Humans

Isoflavones are considered chemoprotective [133] and can be used as an alternative therapy for a wide range of hormonal disorders, including several cancer types, namely breast cancer and prostate cancer [134,135,136], cardiovascular diseases [137,138,139], osteoporosis [140], or menopausal symptoms [141,142].

Since Setchell et al. [20] presented the hypothesis that the clinical effectiveness of the soybean isoflavones may be a function of the ability to transform them to a more potent estrogenic metabolite, the research interest is focused primarily on equol. In vitro studies proved that equol is more bioactive, more estrogenic [104], and a more potent antioxidant [143] than the source form daidzein. Equol also demonstrated anti-androgenic properties [144]. Apart from that, the free effective equol levels circulating in the human serum are higher [145] and its degradation in plasma is slower when compared to daidzein [20].

On the other hand, isoflavones may also be considered endocrine disruptors with possible negative influences on the state of health in a certain part of population [45,116,146], or on the environment [52].

**Figure 1:**
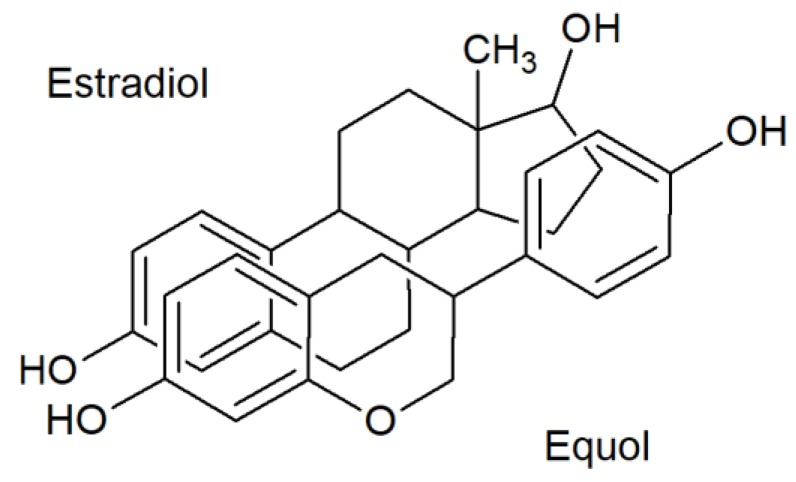
Comparison of estrogen and isoflavone chemical structures [101] Copyright number: 4533100249172 from Oxford University Press.

Although the chemical structure of isoflavones is different from that of endogenous estrogens, their common feature is a phenol group, which enables their attachment to, and activation of, estrogen receptors (ER, Figure 1) [147]. In the cell nuclei of target tissues, isoflavones bind to ER and regulate gene expression. However, their retention time in the cell nucleus is short. The isoflavone affinity to β-ER isoforms is approximately five times higher than the affinity to α-ER isoforms [148], in contrast to estradiol (E2), whose affinities to both receptor types are roughly the same [134]. The β-ER are situated predominantly in the bones, lungs, prostate, urinary bladder, skin, and brain, while the α-ER are situated largely in the mammary gland, testes, uterus, kidneys, and hypophysis [149].

Based on in vitro and in vivo studies of the phytoestrogen potency, the following rank order of the estrogenic potency was estimated: estradiol > genistein and equol > glycitein > daidzein > formononetin and biochanin A [19]. In relation to estrogens, phytoestrogens behave as agonists, or, in high concentrations, as antagonists, which, binding to the ER, block the endogenous estrogen effects [31]. In case of estrogen deficit, they act as weak estrogens [150]. In comparison to estradiol, phytoestrogens show lower estrogenic potency. However, we must take into account that phytoestrogens may be consumed in large amounts. This may lead to a phytoestrogen level several times higher than the level of endogenous estrogens and to the compensation of their lower affinity to ER [77].

### 8.1. Menopausal Symptoms and Estrogenic Activity

Concerns about potential negative effects of hormonal substitution therapy used for the treatment of menopausal symptoms brought the phytoestrogen dietary supplements into focus. [142]. Menopausal women may thus be exposed to high isoflavone doses [141]. Even though many studies report the ability of genistein supplements to reduce menopausal symptoms [141], the results are ambiguous [151]. Generally, taking the dietary supplements that contain isoflavones leads to a modest reduction in the frequency of hot flashes (10–20%). Stronger isoflavone effects were noticed by women with higher frequency of the flashes [152]. Jou et al. [153] determined that only women able to produce equol (detected in the urine) noticed a reduction in menopausal symptoms when taking soybean isoflavone supplements.

Even though phytoestrogens seem to have a positive impact on post-menopausal women, their effect might be harmful to women in the reproductive age. In these women, isoflavones may cause menstrual cycle disorders (dysmenorrhea), endometriosis, and secondary infertility [154]. These problems are reduced, or disappear, when soybean and soybean products are excluded from the diet.

### 8.2. Cardiovascular Diseases

In the population that consumes high amounts of soy products, a lower incidence of heart diseases was reported [137]. Even though there are many risk factors associated with cardiovascular diseases, the main factors are lipid abnormalities [155]. Low density lipoproteins (LDL) penetrate the blood vessel walls, where they are oxidized by free radicals. LDL then accumulate and plug the blood vessels, thereby causing thrombosis. The soy protein was found to lower LDL cholesterol [139]. Zhan and Ho [156] and Sacks et al. [157] assume that soy protein that contains isoflavones is more effective in reducing the LDL cholesterol than isoflavone-depleted soy protein or extracted soybean isoflavones. On the other hand, according to Reynolds et al. [158], the positive effect of isoflavones on the LDL cholesterol levels is not dependent on the soy protein.

Maintaining normal arterial function plays an important role in the prevention of cardiovascular diseases. The vessel ability to dilate in reaction to nitric oxide produced by endothelial cells that skirt the inner surface of the blood cells is lowered in people with a high risk of cardiovascular diseases [159]. The blood vessel endothelium was found to be rich in β-ER, which are preferred as binding partners by isoflavones [160]. However, results of the studies conducted so far are ambiguous. In post-menopausal women taking up to 80 mg of soy isoflavones per day [161], or up to 60 g of the soy protein per day [162], the vasodilatation ability of the blood vessels was not improved. By contrast, another study reported that in post-menopausal women with a daily intake of 80 mg of soy isoflavone extract for five weeks, as well as in men and post-menopausal women taking 40 g of soy protein (i.e., 118 mg of isoflavones) for three months, the arterial stiffness was significantly reduced [138]. However, a recent observation in subjects with hypertension taking the same daily amount of isoflavones for six months did not prove any improvement of the arterial function [163].

Liu et al. [164] concluded from a meta-analysis of available data that a daily consumption of 65–153 mg of soy isoflavones together with the soy protein for 1–12 months lowered the blood pressure in hypertensive population. This effect was not manifested in the population with normal blood pressure.

### 8.3. Bone Health

Osteoporosis is characterized by reduced bone mass and by damaged construction of the bone tissue. It occurs primarily in women and is connected to aging and hormone deficiency [165]. Practical studies on genistein showed a positive effect on osteoporotic bones. Genistein reduces osteoclastic factors (e.g., collagen *C*-telopeptide) and increases osteoblastic factors (e.g., bone alkaline phosphatases). Genistein also selectively antagonizes the catabolic effects of parathormone in the osteoblasts [166]. The mechanism of isoflavone’s effect on osteoclasts probably does not depend on estrogen mechanisms, as there are no ER in the osteoclast nuclei [167].

The results of short-term clinical studies (six months or less) on the effect of increased soybean intake on biochemical markers of bone formation and resorption are ambiguous. Although some studies on post-menopausal women showed that increasing the soy product, soy protein, or soy isoflavone intake improves the markers of bone resorption and formation or reduces bone mass losses [140], other studies did not prove this positive influence of increased soybean intake [168]. Similarly, two long-term clinical studies reported significantly lower bone mass losses in post-menopausal women that took the soy protein containing isoflavones for more than six months than in post-menopausal women that took a corresponding amount of milk protein [169]; however, two other studies on the same dietary supplements (soy protein containing isoflavones and milk protein) did not find any significant differences in the bone mass loss between the groups [170]. Discrepancies in the results were found also in many other studies, i.e., in recent studies on Taiwanese and European post-menopausal women [171,172]. These discrepancies may possibly be explained by the differences in the time of exposure to isoflavones (in the Asian population, the results may be influenced by the lifelong isoflavone intake), or the isoflavone amounts in food (there might be a critical level for the effect manifestation). The biological availability may also differ between particular food supplements, foodstuffs, and pure compounds. Wu et al. [112], Ishimi [173], Taku et al. [174], and others assume that the isoflavone impact on the bone health might depend on the equol-producer status of the subject, as there was a prophylactic effect of the S-equol supplement on the bone-mass reduction in postmenopausal women [175]. Tousen et al. [176] determined that daidzein or equol increased the bone density in growing female rats by the stimulation of bone formation without having a significant impact on the reproductive organ mass. Equol was in this study more effective than daidzein. The bone mass losses were suppressed also in ovariectomized mice [177].

### 8.4. Breast Cancer

Breast cancer is one of the most common terminal diseases in women [178]. The occurrence of the breast cancer in Asia, where the average daily intake of isoflavones reaches 25–50 mg, is lower than in western countries, where the average daily intake of isoflavones is lower than 2 mg [179,180]. The increased soy intake was connected to the risk minimization of the breast cancer formation in two of the four epidemiological studies that examined a broad range of diet components in connection to the breast cancer risk [181,182].

Several studies indicate that a higher isoflavone intake during childhood and/or maturing may lower the risk of the breast cancer formation in later years [135]. Shu et al. [136] proved that in women that fought off breast cancer, the consumption of foods rich in soy isoflavones lowered the death risk by 29% and the risk of the cancer recurrence by 32%.

A considerably higher α-ER/β-ER ratio is connected to some breast cancer types, when compared to a healthy tissue [183], namely because of a reduction in the β-ER level [184]. α-ER activation was proved to stimulate cell proliferation in the breast tissue, while β-ER participate in proliferation inhibition and apoptosis stimulation [183,185]. Isoflavones show a higher affinity to the β-ER [185]. According to Islam et al. [186], isoflavone impact on the inhibition or activation of cell proliferation depends on the specific ratio between α-ER and β-ER in the cells. Isoflavone intake during childhood or maturing contributes to the breast tissue differentiation, which leads to a reduction in anatomical structures that result in the cancer cell formation.

### 8.5. Uterine Cancer

In the case of the uterine cancer, the assumption that a long-term imbalance in estrogen and progesterone levels contributes significantly to cancer formation is generally accepted. Therefore, high isoflavone dosage providing antiestrogenic activity might be a prophylactic against endometrial carcinoma [187]. But again, the results of available studies are ambiguous. While Xu et al. [188] reported a lower isoflavone intake in women suffering from this cancer type as compared to healthy women, Murray et al. [189] state that in postmenopausal women, the daily isoflavone supplementation of 120 mg in six months did not prevent endometrial hyperplasia, which was caused by exogenous estradiol administration.

### 8.6. Prostate Cancer

Prostate cancer mortality is considerably higher in the USA than in Asian countries like Japan or China [190]. Results of cell culture experiments and animal studies indicate a potential role of soybean isoflavones in the limitation of the prostate cancer progression [191]. Although administration of a soy isoflavone supplement for up to one year did not significantly lower the prostate-specific antigen (PSA) in the blood serum of men that had not confirmed prostate cancer yet [192], the soy isoflavone supplement seems to slow down rising concentration of PSA in the blood serum, a phenomenon that is connected to prostate tumor growth in patients with prostate cancer [193]. Nevertheless, epidemiological studies do not provide any clear evidence of the connection between a high isoflavone intake and a low risk of the prostate cancer formation. Messina et al. [134] state that in patients with the prostate cancer, the isoflavone supplement influenced the PSA concentration favorably in four from eight studies. Yan and Spitznagel [194] conclude from a meta-analysis of eight studies that isoflavone consumption was connected to a lowered risk of prostate cancer formation.

### 8.7. Thyroid Function

In experiments performed on cell cultures and on animals, soybean isoflavones have been proven to inhibit thyroid peroxidase activity, the enzyme that is needed for the thyroid hormone synthesis [195]. However, even a high isoflavone intake does not seem to increase the risk of hypothyroidism, as long as an appropriate iodine intake is provided [196]. This is supported by the fact that since iodine is put in the soy-based infant formula, no other hypothyroidism cases have been described in the soy-based formula fed to children [197]. Similar findings were described also in studies on pre- and postmenopausal women with a sufficient iodine intake. In these women, no negative influence of high isoflavone consumption on the levels of circulating thyroid hormones was found [198].

Genistein and, to a lower extent, daidzein compete with thyroxine in the attachment to transthyretin (in vitro study) [199], the main transport protein for thyroid hormones [200]. Displacing of thyroxine from transthyretin may lead to an increased free thyroxine level and subsequently to a change of the thyroid hormone homeostasis [201]. Nevertheless, these negative effects did not manifest when sufficient iodine amount was provided [195].

### 8.8. Isoflavones as Prophylactics Against Irradiation

Recent in vitro (cell cultures) and in vivo (mice) experiments conducted by Hilmann et al. [202] proved that soybean administration before, during, and after the radiotherapy may increase the efficacy of the therapy against the target tumor and at the same time lower the toxicity of the radiotherapy dosage to the surrounding lung tissue. The research in this field, however, is in its infancy.

### 8.9. Antioxidant Activity

Apart from the estrogen activity, isoflavones exhibit a considerable antioxidant activity, which is independent from their estrogenic properties [203]. The antioxidant activity requires the presence of two hydroxyl groups (in the C-4 and C-7 positions), like the ones of daidzein. Aglycons show higher activity than glycosides [22]. The isoflavone antioxidant activity was proved both in vitro [204] and *in vivo*. For example, increased capacity of antioxidant enzymes was described in the mouse epidermis and small intestine following genistein administration [205] and in rats following isoflavone administration [206]. The positive influence of isoflavones on the antioxidant abilities of an organism were described also in several human in vivo studies, but the results are ambiguous. Wiseman et al. [207] found out that the levels of F2-isoprostane and other lipid peroxidation markers were lower after a two-week administration of a soy protein containing 56 mg of isoflavones than in the case of a protein containing only 2 mg of isoflavones. On the other hand, Djuric et al. [208] state that a daily soybean isoflavone dosage of 50 to 100 mg did not exert any significant effect on the level of F2-isoprostanone in the blood plasma or in the urine.

## 9. Isoflavone Utilization for Functional Food Production

Equol is a microbial metabolite of daidzein and formononetin with many beneficial effects on human health (see Section 5, Section 6 and Section 7). It is produced by specific intestinal bacteria of animals and humans [103]. Due to the absence of this specific bacterial population in the gastrointestinal tract of a certain part of the human population [103], taking advantage of equol health benefits is limited.

Intestinal wall inoculation with bacteria transforming daidzein into equol using prebiotics or probiotics, the aim being to induce the equol production in the intestine of adults, has not been successful so far [209]. Oral equol administration, on the other hand, has been proven in several studies as a suitable alternative [20]. Equol produced from daidzein by bacterial strain *Lactococcus garvieae* is usable for the food supplement production, like for example SE5-OH, rich in S-equol. The dose of S-equol contained in one tablet of SE5-OH is 5 mg. Acute and subacute toxicity studies have shown the suplement to be safe [210].

Foodstuffs enriched with isoflavones represent another possibility. From the whole range of foodstuffs commonly consumed by humans, cow milk is probably the only food that can under certain conditions contain relevant amounts of equol [35,54,56,62]. Table 2 summarizes the levels of isoflavones and equol in milk published in the literature.

When consuming one glass of milk (200 mL), according to Table 2, the amount of consumed equol is in the range of 0.7–200.6 µg. Table 2 shows that the highest equol concentrations in milk were obtained in the cases where the source of isoflavones was clover-based. Some other dairy products may also contain isoflavones, although in lower levels. Kuhnle et al. [216] found low equol levels in many commercially available dairy products, except of butter. Recent research showed that the isoflavone concentration in dairy products may be influenced by milk processing. While the heat treatment of milk (up to 140 °C during 20 s) did not influence the isoflavone content [55,59], a part of isoflavones was released to the whey during cheese production, and a decrease in isoflavone levels was detected during cheese ripening [60]. There were changes in the isoflavone contents during the yogurt production (fermentation) and maturation, too [59,217], whereas the equol concentration remained unaffected [217]. These losses might be caused by isoflavone conjugations to proteins or other compounds naturally occurring in the dairy products [217].

## 10. Potential Risks for Humans and Environment Related to Isoflavones

### 10.1. Risks for Children

The prenatal and early postnatal development is one of the most sensitive periods in human life [218]. Under normal circumstances, the exposure of the organism to soybean isoflavones is limited, as genistein and daidzein occurrence in the breast milk is very low (5–15 ng/mL) [219]. A prenatal exposition may occur due to the mother’s way of life (e.g., vegetarian diet, food supplement taking, soy milk consumption) [109], when isoflavones cross the placental barrier and get to the fetal circulation [220].

If the breast-feeding mother consumes food made from soybean, the isoflavone concentration in the breast milk may rise up to ten times [219]. However, even in this case, the nursling daily isoflavone intake from the breast milk reaches only 5–10 µg [108]. The postnatal exposure to isoflavones is therefore connected primarily to the soy-based infant formulae usage, soy milk consumption, or taking children’s food supplements containing soybean [109,221]. In this case, children may be exposed to higher isoflavone levels than adults. Evidence for this statement is given by Setchell et al. [108], who found out that children consuming soy-based infant formulae and foods ingest 6–9 mg of isoflavones per kg of body weight daily, while adults, who consume an adequate amount of soy products, ingest only 1 mg of isoflavones per kg of body weight daily [108]. Moreover, the isoflavone ʽapparent bioavailabilityʼ was found to be higher in children of diverse age than in adults [116]. Irvine et al. [222] determined that 2–16 weeks old infants that were fed soy-based infant formulae excrete detectable amounts of daidzein and genistein in the urine and are, therefore, able to absorb and excrete isoflavones from the infant formulae to an extent similar to that of adults [223]. Franke et al. [116] report even higher isoflavone excretion in children as compared to adults, when consuming the same soybean amounts per kg of body weight.

Due to the low estrogen levels and incomplete development of the hypothalamic-pituitary-ovarian axis, children are more sensitive than adults to exogenic estrogen compounds [224]. Isoflavone exposure during intrauterine development may influence the reproductive system development in female fetuses [225]. Soybean isoflavones might, therefore, constitute a potential risk for the healthy development of the reproductive system. Studies on model animals [226,227] proved that an intrauterine isoflavone exposure may influence the reproductive system function during adulthood [228]. A lowered sensitivity of the mammary gland to estrogens [229] and changes in the ratio of estrogen receptors, leading to influencing of the physiological estrogen effect and to changes in the uterus and ovary development, were detected [230]. Additionally, unlike the exposure in adulthood, the pre- or perinatal exposition may lead to irreversible changes in the reproductive system [146]. Genistein administration during the fetal period increased the risk of uterine cancer development [231]. Isoflavone exposure during prenatal and postnatal development caused changes in the testis epithelium morphology in rats. Higher estradiol levels and lower testosterone levels were also observed in the blood plasma [232].

Conducting studies on nurslings during their postnatal development is virtually impossible due to ethical reasons; therefore, the results of studies on laboratory animals are taken as the base. For example, a recent study of Wang et al. [233] showed that low doses of soybean isoflavones administered from weaning until sexual maturity influenced the follicular development in the ovaries of rats, so that both the follicular atresia and the CL number increased, and a low estradiol level was found in the blood serum. Speeding up the follicular atresia is considered to be the main cause of premature failure of the ovary function [234]. Using metabolomic analysis, changes in levels of 24 metabolites, including sexual hormones, amino acids, fatty acids, and metabolites involved in energy metabolism, were detected in the follicular fluid [233]. The energy metabolism disruption may consequently lead to a damage of mitochondria structure and function, which is the signal of apoptosis activation [235]. Furthermore, recent studies showed that daidzein and genistein can inhibit the cancer cell growth by apoptosis activation [236,237]. Wang et al. [238] hypothesize that the isoflavone exposure from weaning until sexual maturity may influence the follicular development by setting of apoptosis through the apoptotic signaling pathway activation. On the other hand, Strom et al. [239] did not find any isoflavone-adverse effects in women that were administered soy-based infant formulae during early childhood, as compared to the infant formulae made from bovine milk. Likewise, many other studies that compared the infant formulae based on bovine milk and on soybean did not show any differences in growth and development of nurslings [240], in their body mass and length [241], or in the size of sexual-hormone sensitive organs [242].

### 10.2. Estrogen Activity of Animal Farming Waste

Although the equol-enriched bovine milk production seems to be desirable from the human-nutrition point of view, the estrogen character of equol cannot be ignored, as it is excreted in surplus also in the urine and feces of the dairy cows. The urinary and fecal equol may, thus, become a potential resource of environmental pollution. Present studies concerning the residues of steroidal estrogens occurring naturally in the animal farming waste [243,244] did not monitor the involvement of equol, or generally of dietary phytoestrogens, in the estrogen activity, although they are considered a potential source of estrogenic contamination [245]. Among available data, the study of Hoerger et al. [246] can be mentioned, who found inland Swiss rivers to be loaded with several kilograms of isoflavones and equol per year, mainly during summer. Formononetin and equol were detected most often, in amounts reaching 217 and 524 ng/L, respectively [247]. Likewise, Kuster et al. [248] found up to 366 ng/L of phytoestrogens in the waters of the Rio de Janeiro region (Brazil). The estimation of dietary isoflavone involvement in the environmental estrogenic contamination requires further study.

## 11. Conclusions

Isoflavones are polyphenolic compounds that represent one of the most common categories of phytoestrogens. These secondary plant metabolities are structurally similar to 17β-estradiol and are found mainly in the *Fabaceae* family usually in a conjugated form. Before being metabolized, they are hydrolysed into aglycones by the microflora present in the human or animal digestive tract or by the enzymes of the gastrointestinal tract.

Except for their role in the plant interaction with its environment, they also show numerous health benefits often connected with their estrogenic activity, for which they have been intensively studied for decades. On the other hand, they may also be considered endocrine disruptors with the potential to cause adverse effects on human or animal health either directly through their consumption in food/feed or indirectly through the contamination of the environment, mainly surface water.

Furthermore, inconsistency in the results obtained from isoflavone studies performed on various subjects suggest that microflora involved in the metabolism of isoflavones plays a key role in their final effect on the target organism because it can convert the parent molecules into metabolites, such as equol, with altered (higher) estrogenic activity. Many studies focused on identification of bacteria capable to convert daidzein into equol with the aim to produce food supplements, while other studies focused on production of isoflavone/equol-enriched milk or dairy products through the manipulation of dairy diets and metabolic processes in the rumen. However, these manipulations lead to increased excretion of isoflavones into the environment with subsequent estrogenic contamination of surface water. Further studies in many areas of isoflavone research are needed to improve our understanding in this extremely complex field of study.

## Figures and Tables

**Table 1:** The structure of isoflavones and their glycosides [24,25].

**Aglycon**	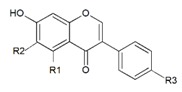	**R1**	**R2**	**R3**	
Daidzein		H	H	OH	
Genistein		OH	H	OH	
Glycitein		H	OCH_3_	OH	
Formononetin		H	H	OCH_3_	
Biochanin A		OH	H	OCH_3_	
**Glucoside**	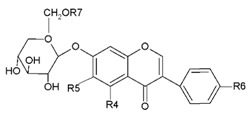	**R4**	**R5**	**R6**	**R7**
Daidzin		H	H	OH	H
Genistin		H	H	OH	H
Glycitin		H	OCH_3_	OH	H
Ononin		H	H	OCH_3_	H
Sissotrin		OH	H	OCH_3_	H
Acetyldaidzin		H	H	OH	COCH_3_
Acetylgenistin		OH	H	OH	COCH_3_
Acetylglycitin		H	OCH_3_	OH	COCH_3_
Malonyldaidzin		H	H	OH	COCH_2_COOH
Malonylgenistin		OH	H	OH	COCH_2_COOH
Malonylglycitin		H	OCH_3_	OH	COCH_2_COOH
Malonylononin		H	H	OCH_3_	COCH_2_COOH
Malonylsissotrin		OH	H	OCH_3_	COCH_2_COOH

**Table 2:** Equol and isoflavone concentrations in bovine milk samples.

Analyte		Values	Isoflavone Source	Reference
Daidzein	µg/L	0.3–1.9	C	[211]
		0.8–2.1	C	[62]
		0.3–5.7	C	[212]
		0.2–7.7	C	[35]
		0.0–9.6	U	[213]
		12.5–15.6	S	[53]
		10–19	S	[214]
		36.5–40.3	S	[59]
		5.6–112.4	S	[61]
Equol	µg/L	3.6–15.6	S	[59]
		3.5–54.8	S	[53]
		4–76	S	[214]
		14.5–61.1 *	S	[58]
		11–130	U	[215]
		171–287	C	[54]
		14.1–293.0	U	[213]
		34.6–340.2	S	[61]
		35–345	C	[55]
		18.8–355.4	C	[211]
		52–364	C	[35]
		57–1003	C + A	[212]
Genistein	µg/L	1.7–2.2	C	[62]
		1.9–3.0	C	[35]
		0.0–5.8	U	[213]
		2.4–31.3	S	[61]
		3–37	C	[55]
		34.4–37.3	S	[53]
		170.6–175.8	S	[59]
Glycitein	µg/L	3.6–4.5	C	[211]
		23.4–27.9	S	[59]
		0.0–189.1	S	[61]

C: clover, S: soybeans, C + A: clover and alfalfa, U: unspecified, commercial milks, * concentration of equol expressed in µg/kg.

## References

[1] Kurzer M.S., Xu X. (1997). Dietary phytoestrogens. Annu. Rev. Nutr..

[2] Bennetts H.W., Uuderwood E.J., Shier F.L. (1946). A specific breeding problem of sheep on subterranean clover pastures in Western Australia. Aust. Vet. J..

[3] Mustonen E., Taponen S., Andersson M., Sukura A., Katila T., Taponen J. (2014). Fertility and growth of nulliparous ewes after feeding red clover silage with high phyto-oestrogen concentrations. Animal.

[4] Lightfoot R.J., Croker K.P., Neil H.G. (1968). Failure of sperm transport in relation to ewe infertility following prolonged grazing on oestrogenic pastures. Aust. J. Agric. Res..

[5] Rossiter R.C., Beck A.B. (1966). Physiological and ecological studies on the estrogenic isoflavones in subterranean clover (Trifolium subterraneum) I. Effects of temperature. Aust. J. Agric. Res..

[6] Braden A., Hart N., Lamberton J. (1967). The estrogenic activity and metabolism of certain isoflavones in sheep. Aust. J. Agric. Res..

[7] Nottle M.C. (1976). Composition of some urinary calculi of ruminants in Western Australia. Res. Vet. Sci..

[8] Marrian G.F., Haslewood G.A. (1932). Equol, a new inactive phenol isolated from the ketohydroxyoestrin fraction of mares’ urine. Biochem. J..

[9] Marrian G.F., Beall D. (1935). The constitution of equol. Biochem. J..

[10] Shutt D., Braden A. (1968). The significance of equol in relation to the oestrogenic responses in sheep ingesting clover with a high formononetin content. Aust. J. Agric. Res..

[11] Shutt D.A., Weston R.H., Hogan J.P. (1970). Quantitative aspects of phytoestrogen metabolism in sheep fed on subterranean clover (Trifolium subterraneum, cultivar Clare) or red clover (*Trifolium pratense*). Aust. J. Agric. Res..

[12] Lundh T. (1995). Metabolism of Estrogenic Isoflavones in Domestic Animals. Proc. Soc. Exp. Biol. Med..

[13] Klyne W., Wright A.A. (1959). Steroids and other lipids of pregnant cow’s urine. J. Endocrinol..

[14] Chang H.H.-S., Robinson A.R., Common R.H. (1975). Excretion of Radioactive Daidzein and Equol as Monosulfates and Disulfates in the Urine of the Laying Hen. Can. J. Biochem..

[15] Blair R.M., Appt S.E., Franke A.A., Clarkson T.B. (2003). Treatment with antibiotics reduces plasma equol concentration in cynomolgus monkeys (Macaca fascicularis). J. Nutr..

[16] Brown N.M., Setchell K.D. (2001). Animal models impacted by phytoestrogens in commercial chow: Implications for pathways influenced by hormones. Lab. Investig..

[17] Juniewicz P.E., Morell S.P., Moser A., Ewing L.L. (1988). Identification of phytoestrogens in the urine of male dogs. J. Steroid Biochem..

[18] Axelson M., Kirk D.N., Farrant R.D., Cooley G., Lawson A.M., Setchell K.D. (1982). The identification of the weak oestrogen equol [7-hydroxy-3-(4’-hydroxyphenyl)chroman] in human urine. Biochem. J..

[19] Committee on Toxicity (2003). Phytoestrogens and Health: COT Report. https://cot.food.gov.uk/sites/default/files/cot/phytoreport0503.pdf.

[20] Setchell K.D.R., Brown N.M., Lydeking-Olsen E. (2002). The clinical importance of the metabolite equol-a clue to the effectiveness of soy and its isoflavones. J. Nutr..

[21] Dixon R.A. (2003). Legume Natural Products: Understanding and Manipulating Complex Pathways for Human and Animal Health. Plant Physiol..

[22] Ko K.-P. (2014). Isoflavones: Chemistry, Analysis, Functions and Effects on Health and Cancer. Asian Pac. J. Cancer Prev..

[23] Coward L., Barnes N.C., Setchell K.D.R., Barnes S. (1993). Genistein, daidzein, and their β-glycoside conjugates: Antitumor isoflavones in soybean foods from American and Asian diets. J. Agric. Food Chem..

[24] Bingham S.A., Atkinson C., Liggins J., Bluck L., Coward A. (1998). Phyto-oestrogens: Where are we now?. Br. J. Nutr..

[25] Daems F., Romnee J.-M., Heuskin S., Froidmont É., Lognay G. (2016). Analytical methods used to quantify isoflavones in cow’s milk: A review. Dairy Sci. Technol..

[26] Dakora F.D., Phillips D.A. (1996). Diverse functions of isoflavonoids in legumes transcend anti-microbial definitions of phytoalexins. Physiol. Mol. Plant Pathol..

[27] Bellou S., Karali E., Bagli E., Al-Maharik N., Morbidelli L., Ziche M., Adlercreutz H., Murphy C., Fotsis T. (2012). The isoflavone metabolite 6-methoxyequol inhibits angiogenesis and suppresses tumor growth. Mol. Cancer.

[28] Rípodas C., Via V.D., Aguilar O.M., Zanetti M.E., Blanco F.A. (2013). Knock-down of a member of the isoflavone reductase gene family impairs plant growth and nodulation in Phaseolus vulgaris. Plant Physiol. Biochem..

[29] Subramanian S., Stacey G., Yu O. (2006). Endogenous isoflavones are essential for the establishment of symbiosis between soybean and Bradyrhizobium japonicum. Plant J..

[30] Sukumaran A., McDowell T., Chen L., Renaud J., Dhaubhadel S. (2018). Isoflavonoid-specific prenyltransferase gene family in soybean: GmPT01, a pterocarpan 2-dimethylallyltransferase involved in glyceollin biosynthesis. Plant J..

[31] Liu Y., Hassan S., Kidd B.N., Garg G., Mathesius U., Singh K.B., Anderson J.P. (2017). Ethylene Signaling Is Important for Isoflavonoid-Mediated Resistance to Rhizoctonia solani in Roots of Medicago truncatula. Mol. Plant-Microbe Interact..

[32] Hasanah Y., Nisa T.C., Armidin H., Hanum H. (2015). Isoflavone content of soybean [*Glycine max* (L). *Merr*.] cultivars with different nitrogen sources and growing season under dry land conditions. JAEID.

[33] Adler S.A., Purup S., Hansen-Møller J., Thuen E., Steinshamn H. (2015). Phytoestrogens and Their Metabolites in Bulk-Tank Milk: Effects of Farm Management and Season. PLoS ONE.

[34] Saloniemi H., Wähälä K., Nykanen-Kurki P., Kallela K., Saastamoinen I. (1995). Phytoestrogen Content and Estrogenic Effect of Legume Fodder. Exp. Biol. Med..

[35] Steinshamn H., Purup S., Thuen E., Hansen-Møller J. (2008). Effects of Clover-Grass Silages and Concentrate Supplementation on the Content of Phytoestrogens in Dairy Cow Milk. J. Dairy Sci..

[36] Butkutė B., Padarauskas A., Cesevičienė J., Taujenis L., Norkevičienė E. (2018). Phytochemical composition of temperate perennial legumes. Crop Pasture Sci..

[37] Rizzo G., Baroni L. (2018). Soy, Soy Foods and Their Role in Vegetarian Diets. Nutrients.

[38] Bustamante-Rangel M., Delgado-Zamarreño M.M., Pérez-Martín L., Rodríguez-Gonzalo E., Domínguez-Álvarez J. (2018). Analysis of Isoflavones in Foods: Analysis of isoflavones in foods…. Compr. Rev. Food Sci. Food Saf..

[39] Frankenfeld C.L. (2011). Dairy consumption is a significant correlate of urinary equol concentration in a representative sample of US adults. Am. J. Clin. Nutr..

[40] Andres S., Hansen U., Niemann B., Palavinskas R., Lampen A. (2015). Determination of the isoflavone composition and estrogenic activity of commercial dietary supplements based on soy or red clover. Food Funct..

[41] Klyne W., Wright A.A. (1957). Steroids and other lipids of pregnant goat’s urine. Biochem. J..

[42] Miksicek R.J. (1995). Estrogenic Flavonoids: Structural Requirements for Biological Activity. Exp. Biol. Med..

[43] Nilsson A., Hill J.L., Davies H.L. (1967). An in vitro study of formononetin and biochanin A in rumen fluid from sheep. Biochim. Biophys. Acta.

[44] Dickinson J.M., Smith G.R., Randel R.D., Pemberton I.J. (1988). In vitro metabolism of formononetin and biochanin A in bovine rumen fluid. J. Anim. Sci..

[45] Wocławek-Potocka I., Mannelli C., Boruszewska D., Kowalczyk-Zieba I., Waśniewski T., Skarżyński D.J. (2013). Diverse Effects of Phytoestrogens on the Reproductive Performance: Cow as a Model. Inter. J. Endocrinol..

[46] Choi E.J., Kim G.-H. (2014). The antioxidant activity of daidzein metabolites, O-desmethylangolensin and equol, in HepG2 cells. Mol. Med. Rep..

[47] Njåstad K.M., Adler S.A., Hansen-Møller J., Thuen E., Gustavsson A.-M., Steinshamn H. (2014). Gastrointestinal metabolism of phytoestrogens in lactating dairy cows fed silages with different botanical composition. J. Dairy Sci..

[48] Adams N.R. (1995). Detection of the effects of phytoestrogens on sheep and cattle. J. Anim. Sci..

[49] Trnková A., Šancová K., Zapletalová M., Kašparovská J., Dadáková K., Křížová L., Lochman J., Hadrová S., Ihnatová I., Kašparovský T. (2018). Determination of in vitro isoflavone degradation in rumen fluid. J. Dairy Sci..

[50] Lundh T.J.O., Pettersson H.I., Martinsson K.A. (1990). Comparative levels of free and conjugated plant estrogens in blood plasma of sheep and cattle fed estrogenic silage. J. Agric. Food Chem..

[51] Urpi-Sarda M., Morand C., Besson C., Kraft G., Viala D., Scalbert A., Besle J.-M., Manach C. (2008). Tissue distribution of isoflavones in ewes after consumption of red clover silage. Arch. Biochem. Biophys..

[52] Tucker H.A., Knowlton K.F., Meyer M.T., Khunjar W.O., Love N.G. (2010). Effect of diet on fecal and urinary estrogenic activity. J. Dairy Sci..

[53] Třináctý J., Křížová L., Schulzová V., Hajšlová J., Hanuš O. (2009). The effect of feeding soybean-derived phytoestogens on their concentration in plasma and milk of lactating dairy cows. Arch. Anim. Nutr..

[54] Mustonen E.A., Tuori M., Saastamoinen I., Taponen J., Wähälä K., Saloniemi H., Vanhatalo A. (2009). Equol in milk of dairy cows is derived from forage legumes such as red clover. Br. J. Nutr..

[55] King R.A., Mano M.M., Head R.J. (1998). Assessment of isoflavonoid concentrations in Australian bovine milk samples. J. Dairy Res..

[56] Höjer A., Adler S., Purup S., Hansen-Møller J., Martinsson K., Steinshamn H., Gustavsson A.-M. (2012). Effects of feeding dairy cows different legume-grass silages on milk phytoestrogen concentration. J. Dairy Sci..

[57] Sakakibara H., Viala D., Doreau M., Besle J.-M. Clover isoflavones move to cows’ milk. Proceedings of the 1st International Conference on Polyphenols and Health.

[58] Flachowsky G., Hünerberg M., Meyer U., Kammerer D.R., Carle R., Goerke M., Eklund M. (2011). Isoflavone concentration of soybean meal from various origins and transfer of isoflavones into milk of dairy cows. J. Verbrauch. Lebensm..

[59] Křížová L., Třináctý J., Hajšlová J., Havlíková Š., Ng T.-B. (2011). The Effect of Technological Processing on the Content of Isoflavones in Bovine Milk and Dairy Products. Soybean—Applications and Technology.

[60] Křížová L., Veselý A., Třináctý J., Schulzová V., Hurajová A., Hajšlová J., Kvasničková E., Havlíková Š. (2011). Changes in isoflavones concentrations in cheese during processing and ripening. Acta Univ. Agric. Silvic. Mendel. Brun..

[61] Kasparovska J., Pecinkova M., Dadakova K., Krizova L., Hadrova S., Lexa M., Lochman J., Kasparovsky T. (2016). Effects of Isoflavone-Enriched Feed on the Rumen Microbiota in Dairy Cows. PLoS ONE.

[62] Andersen C., Weisbjerg M.R., Hansen-Møller J., Sejrsen K. (2009). Effect of forage on the content of phyto-oestrogens in bovine milk. Animal.

[63] Turner C.W. (1958). Estrogen Content of Colostrum and Milk of Dairy Cattle. J. Dairy Sci..

[64] Shennan D.B., Peaker M. (2000). Transport of milk constituents by the mammary gland. Physiol. Rev..

[65] Schwen R.J., Nguyen L., Jackson R.L. (2012). Elucidation of the metabolic pathway of S-equol in rat, monkey and man. Food Chem. Toxicol..

[66] Whitehouse-Tedd K.M., Cave N.J., Ugarte C.E., Waldron L.A., Prasain J.K., Arabshahi A., Barnes S., Thomas D.G. (2011). Dietary isoflavone absorption, excretion, and metabolism in captive cheetahs (*Acinonyx jubatus*). J. Zoo Wildl. Med..

[67] Marshall T. (1973). Clover disease: What do we know and what can we do. J. Dep. Agric. West. Aust. Ser. 4.

[68] Sakakibara H., Viala D., Ollier A., Combeau A., Besle J.-M. (2004). Isoflavones in several clover species and in milk from goats fed clovers. Biofactors.

[69] Woclawek-Potocka I., Bah M.M., Korzekwa A., Piskula M.K., Wiczkowski W., Depta A., Skarzynski D.J. (2005). Soybean-derived phytoestrogens regulate prostaglandin secretion in endometrium during cattle estrous cycle and early pregnancy. Exp. Biol. Med..

[70] Woclawek-Potocka I., Borkowski K., Korzekwa A., Okuda K., Skarzynski D.J. (2006). Phyto- and endogenous estrogens differently activate intracellular calcium ion mobilization in bovine endometrial cells. J. Reprod. Dev..

[71] Woclawek-Potocka I., Piskula M.K., Bah M., Siemieniuch M.J., Korzekwa A., Brzezicka E., Skarzynski D.J. (2008). Concentrations of isoflavones and their metabolites in the blood of pregnant and non-pregnant heifers fed soy bean. J. Reprod. Dev..

[72] Piotrowska K.K., Woclawek-Potocka I., Bah M.M., Piskula M.K., Pilawski W., Bober A., Skarzynski D.J. (2006). Phytoestrogens and their metabolites inhibit the sensitivity of the bovine corpus luteum to luteotropic factors. J. Reprod. Dev..

[73] Watzková J., Křížová L., Pavlík A., Schulzová V., Hajšlová J., Lojza J. (2010). The Effect of Soybean-Derived Phytoestrogens on Concentrations of Plasma Isoflavones, 15-keto-13,14-dihydroprostaglandin F2α and Progesterone in Dairy Cows. Acta Vet. Brno.

[74] Goff A.K. (2004). Steroid hormone modulation of prostaglandin secretion in the ruminant endometrium during the estrous cycle. Biol. Reprod..

[75] Asselin E., Goff A.K., Bergeron H., Fortier M.A. (1996). Influence of sex steroids on the production of prostaglandins F_2α_ and E_2_ and response to oxytocin in cultured epithelial and stromal cells of the bovine endometrium. Biol. Reprod..

[76] Okuda K., Miyamoto Y., Skarzynski D.J. (2002). Regulation of endometrial prostaglandin F(_2α_) synthesis during luteolysis and early pregnancy in cattle. Domest. Anim. Endocrinol..

[77] Woclawek-Potocka I., Bober A., Korzekwa A., Okuda K., Skarzynski D.J. (2006). Equol and para-ethyl-phenol stimulate prostaglandin F_2α_ secretion in bovine corpus luteum: Intracellular mechanisms of action. Prostaglandins Other Lipid Mediat..

[78] Shore L.S., Rios C., Marcus S., Bernstein M., Shemesh M. (1998). Relationship between peripheral estrogen concentrations at insemination and subsequent fetal loss in cattle. Theriogenology.

[79] Kowalczyk-Zieba I., Woclawek-Potocka I., Piskula M.K., Piotrowska-Tomala K.K., Boruszewska D., Bah M.M., Siemieniuch M.J., Skarzynski D.J. (2011). Experimentally induced mastitis and metritis modulate soy bean derived isoflavone biotransformation in dairy cows. Theriogenology.

[80] Mohanty I., Senapati M.R., Jena D., Behera P.C. (2014). Ethnoveterinary importance of herbal galactogogues—A review. Vet. World.

[81] Dewhurst R.J., Fisher W.J., Tweed J.K.S., Wilkins R.J. (2003). Comparison of grass and legume silages for milk production. 1. Production responses with different levels of concentrate. J. Dairy Sci..

[82] Vanhatalo A., Kuoppala K., Toivonen V., Shingfield K.J. (2007). Effects of forage species and stage of maturity on bovine milk fatty acid composition. Eur. J. Lipid Sci. Technol..

[83] Liu D.-Y., He S.-J., Jin E.-H., Liu S.-Q., Tang Y.-G., Li S.-H., Zhong L.-T. (2013). Effect of daidzein on production performance and serum antioxidative function in late lactation cows under heat stress. Livest. Sci..

[84] Moorby J.M., Fraser M.D., Theobald V.J., Wood J.D., Haresign W. (2004). The effect of red clover formononetin content on live-weight gain, carcass characteristics and muscle equol content of finishing lambs. Anim. Sci..

[85] Speijers M.H.M., Fraser M.D., Theobald V.J., Haresign W. (2005). Effects of ensiled forage legumes on performance of store finishing lambs. Anim. Feed Sci. Technol..

[86] Liu H.Y., Zhang C.Q. (2008). Effects of daidzein on messenger ribonucleic Acid expression of gonadotropin receptors in chicken ovarian follicles. Poult. Sci..

[87] Guo-zhen J., Li W. (2014). Effect of Daidzein on Ileum Microflora Biodiversity in Hy-Line Variety Brown Layers. J. Northeast Agric. Univ..

[88] Etxeberria U., Fernández-Quintela A., Milagro F.I., Aguirre L., Martínez J.A., Portillo M.P. (2013). Impact of Polyphenols and Polyphenol-Rich Dietary Sources on Gut Microbiota Composition. J. Agric. Food Chem..

[89] Setchell K.D.R., Brown N.M., Desai P., Zimmer-Nechemias L., Wolfe B.E., Brashear W.T., Kirschner A.S., Cassidy A., Heubi J.E. (2001). Bioavailability of Pure Isoflavones in Healthy Humans and Analysis of Commercial Soy Isoflavone Supplements. J. Nutr..

[90] Heinonen S., Wähälä K., Adlercreutz H. (1999). Identification of Isoflavone Metabolites Dihydrodaidzein, Dihydrogenistein, 6′-OH-O-dma, and cis-4-OH-equol in Human Urine by Gas Chromatography–Mass Spectroscopy Using Authentic Reference Compounds. Anal. Biochem..

[91] Sfakianos J., Coward L., Kirk M., Barnes S. (1997). Intestinal uptake and biliary excretion of the isoflavone genistein in rats. J. Nutr..

[92] Setchell K.D.R., Faughnan M.S., Avades T., Zimmer-Nechemias L., Brown N.B., Wolfe B., Brashear W.T., Desai P., Oldfield M.F., Botting N.P. (2003). Comparing the pharmacokinetics of daidzein and genistein using 13C-labeled tracers in premenopausal women. Am. J. Clin. Nutr..

[93] Hur H.-G., Lay J.O., Beger R.D., Freeman J.P., Rafii F. (2000). Isolation of human intestinal bacteria metabolizing the natural isoflavone glycosides daidzin and genistin. Arch. Microbiol..

[94] Yerramsetty V., Gallaher D.D., Ismail B. (2014). Malonylglucoside Conjugates of Isoflavones Are Much Less Bioavailable Compared with Unconjugated β-Glucosidic Forms in Rats. J. Nutr..

[95] Zubik L., Meydani M. (2003). Bioavailability of soybean isoflavones from aglycone and glucoside forms in American women. Am. J. Clin. Nutr..

[96] Németh K., Plumb G.W., Berrin J.G., Juge N., Jacob R., Naim H.I., Williamson G., Swallow D.L., Kroon P.A. (2003). Deglycosylation by small intestinal epithelial cell β-glucosidases is a critical step in the absorption and metabolism of dietary flavonoid glycosides in humans. Eur. J. Nutr..

[97] Decroos K., Vanhemmens S., Cattoir S., Boon N., Verstraete W. (2005). Isolation and characterisation of an equol-producing mixed microbial culture from a human faecal sample and its activity under gastrointestinal conditions. Arch. Microbiol..

[98] Ronis M.J., Little J.M., Barone G.W., Chen G., Radominska-Pandya A., Badger T.M. (2006). Sulfation of the isoflavones genistein and daidzein in human and rat liver and gastrointestinal tract. J. Med. Food.

[99] Hosoda K., Furuta T., Yokokawa A., Ishii K. (2010). Identification and quantification of daidzein-7-glucuronide-4’-sulfate, genistein-7-glucuronide-4’-sulfate and genistein-4’,7-diglucuronide as major metabolites in human plasma after administration of kinako. Anal. Bioanal. Chem..

[100] Barnes S. (2010). The biochemistry, chemistry and physiology of the isoflavones in soybeans and their food products. Lymphat. Res. Biol..

[101] Setchell K.D., Cassidy A. (1999). Dietary isoflavones: Biological effects and relevance to human health. J. Nutr..

[102] Gaya P., Medina M., Sánchez-Jiménez A., Landete J. (2016). Phytoestrogen Metabolism by Adult Human Gut Microbiota. Molecules.

[103] Setchell K.D.R. (2004). Equol—Origins, actions, and clinical relevance of this specific soy isoflavone metabolite. J. Nutr..

[104] Muthyala R.S., Ju Y.H., Sheng S., Williams L.D., Doerge D.R., Katzenellenbogen B.S., Helferich W.G., Katzenellenbogen J.A. (2004). Equol, a natural estrogenic metabolite from soy isoflavones: Convenient preparation and resolution of R- and S-equols and their differing binding and biological activity through estrogen receptors α and β. Bioorg. Med. Chem..

[105] Toro-Funes N., Morales-Gutiérrez F.J., Veciana-Nogués M.T., Vidal-Carou M.C., Spencer J.P.E., Rodriguez-Mateos A. (2015). The intracellular metabolism of isoflavones in endothelial cells. Food Funct..

[106] Heinonen S.M., Hoikkala A., Wähälä K., Adlercreutz H. (2003). Metabolism of the soy isoflavones daidzein, genistein and glycitein in human subjects. Identification of new metabolites having an intact isoflavonoid skeleton. J. Steroid Biochem. Mol. Biol..

[107] Axelson M., Setchell K.D. (1981). The excretion of lignans in rats—Evidence for an intestinal bacterial source for this new group of compounds. FEBS Lett..

[108] Setchell K.D., Zimmer-Nechemias L., Cai J., Heubi J.E. (1997). Exposure of infants to phyto-oestrogens from soy-based infant formula. Lancet.

[109] Setchell K.D., Zimmer-Nechemias L., Cai J., Heubi J.E. (1998). Isoflavone content of infant formulas and the metabolic fate of these phytoestrogens in early life. Am. J. Clin. Nutr..

[110] Rowland I.R., Wiseman H., Sanders T.A., Adlercreutz H., Bowey E.A. (2000). Interindividual variation in metabolism of soy isoflavones and lignans: Influence of habitual diet on equol production by the gut microflora. Nutr. Cancer.

[111] Braune A., Blaut M. (2018). Evaluation of inter-individual differences in gut bacterial isoflavone bioactivation in humans by PCR-based targeting of genes involved in equol formation. J. Appl. Microbiol..

[112] Wu J., Oka J., Ezaki J., Ohtomo T., Ueno T., Uchiyama S., Toda T., Uehara M., Ishimi Y. (2007). Possible role of equol status in the effects of isoflavone on bone and fat mass in postmenopausal Japanese women: A double-blind, randomized, controlled trial. Menopause.

[113] Frankenfeld C.L., Atkinson C., Thomas W.K., Gonzalez A., Jokela T., Wähälä K., Schwartz S.M., Li S.S., Lampe J.W. (2005). High concordance of daidzein-metabolizing phenotypes in individuals measured 1 to 3 years apart. Br. J. Nutr..

[114] Akaza H., Miyanaga N., Takashima N., Naito S., Hirao Y., Tsukamoto T., Fujioka T., Mori M., Kim W.-J., Song J.M. (2004). Comparisons of percent equol producers between prostate cancer patients and controls: Case-controlled studies of isoflavones in Japanese, Korean and American residents. Jpn. J. Clin. Oncol..

[115] Franke A.A., Lai J.F., Halm B.M., Pagano I., Kono N., Mack W.J., Hodis H.N. (2012). Equol production changes over time in postmenopausal women. J. Nutr. Biochem..

[116] Franke A.A., Lai J.F., Halm B.M. (2014). Absorption, distribution, metabolism, and excretion of isoflavonoids after soy intake. Arch. Biochem. Biophys..

[117] Frankenfeld C.L., McTiernan A., Tworoger S.S., Atkinson C., Thomas W.K., Stanczyk F.Z., Marcovina S.M., Weigle D.S., Weiss N.S., Holt V.L. (2004). Serum steroid hormones, sex hormone-binding globulin concentrations, and urinary hydroxylated estrogen metabolites in post-menopausal women in relation to daidzein-metabolizing phenotypes. J. Steroid Biochem. Mol. Biol..

[118] Liu B., Qin L., Liu A., Uchiyama S., Ueno T., Li X., Wang P. (2010). Prevalence of the equol-producer phenotype and its relationship with dietary isoflavone and serum lipids in healthy Chinese adults. J. Epidemiol..

[119] Setchell K.D.R., Cole S.J. (2006). Method of defining equol-producer status and its frequency among vegetarians. J. Nutr..

[120] Redruello B., Guadamuro L., Cuesta I., Álvarez-Buylla J.R., Mayo B., Delgado S. (2015). A novel UHPLC method for the rapid and simultaneous determination of daidzein, genistein and equol in human urine. J. Chromatogr. B.

[121] Cassidy A. (1991). Plant Oestrogens and Their Relation to Hormonal Status in Women.

[122] Lipovac M., Pfitscher A., Hobiger S., Laschitz T., Imhof M., Chedraui P., Jungbauer A. (2015). Red clover isoflavone metabolite bioavailability is decreased after fructooligosaccharide supplementation. Fitoterapia.

[123] Nielsen I.L.F., Williamson G. (2007). Review of the factors affecting bioavailability of soy isoflavones in humans. Nutr. Cancer.

[124] Cohen L.A., Crespin J.S., Wolper C., Zang E.A., Pittman B., Zhao Z., Holt P.R. (2007). Soy isoflavone intake and estrogen excretion patterns in young women: Effect of probiotic administration. In Vivo.

[125] Elghali S., Mustafa S., Amid M., Manap M.Y.A.B.D., Ismail A., Abas F. (2012). Bioconversion of daidzein to equol by Bifidobacterium breve 15700 and Bifidobacterium longum BB536. J. Funct. Foods.

[126] Shimada Y., Yasuda S., Takahashi M., Hayashi T., Morihiro M., Sato I., Abiru Y., Uchiyama S., Hishigaki H. (2010). Cloning and expression of a novel NADP(H)-dependent daidzein reductase, an enzyme involved in the metabolism of daidzein, from equol-producing Lactococcus strain 20–92. Appl. Environ. Microbiol..

[127] Kim M., Kim S.-I., Han J., Wang X.-L., Song D.-G., Kim S.-U. (2009). Stereospecific Biotransformation of Dihydrodaidzein into (3S)-Equol by the Human Intestinal Bacterium Eggerthella Strain Julong 732. Appl. Environ. Microbiol..

[128] Kim M., Lee J., Han J. (2015). Deglycosylation of isoflavone C-glycosides by newly isolated human intestinal bacteria. J. Sci. Food Agric..

[129] Tamura M., Tsushida T., Shinohara K. (2007). Isolation of an isoflavone-metabolizing, Clostridium-like bacterium, strain TM-40, from human faeces. Anaerobe.

[130] Schoefer L., Mohan R., Braune A., Birringer M., Blaut M. (2002). Anaerobic C-ring cleavage of genistein and daidzein by Eubacterium ramulus. FEMS Microbiol. Lett..

[131] Wang X.-L., Kim H.-J., Kang S.-I., Kim S.-I., Hur H.-G. (2007). Production of phytoestrogen S-equol from daidzein in mixed culture of two anaerobic bacteria. Arch. Microbiol..

[132] Ueno T., Uchiyama S. (2001). Identification of the specific intestinal bacteria capable of metabolising soy isoflavone to equol. (Abs.). Ann. Nutr. Metab..

[133] Baber R.J., Preedy V.R. (2013). Phytoestrogens in health: The role of isoflavones. Isoflavones: Chemistry, Analysis, Function and Effects.

[134] Messina M., Kucuk O., Lampe J.W. (2006). An overview of the health effects of isoflavones with an emphasis on prostate cancer risk and prostate-specific antigen levels. J. AOAC Int..

[135] Messina M., Hilakivi-Clarke L. (2009). Early intake appears to be the key to the proposed protective effects of soy intake against breast cancer. Nutr. Cancer.

[136] Shu X.O., Zheng Y., Cai H., Gu K., Chen Z., Zheng W., Lu W. (2009). Soy food intake and breast cancer survival. JAMA.

[137] Carroll K.K. (1991). Review of clinical studies on cholesterol-lowering response to soy protein. J. Am. Diet. Assoc..

[138] Teede H.J., Dalais F.S., Kotsopoulos D., Liang Y.L., Davis S., McGrath B.P. (2001). Dietary soy has both beneficial and potentially adverse cardiovascular effects: A placebo-controlled study in men and postmenopausal women. J. Clin. Endocrinol. Metab..

[139] Hoie L.H., Guldstrand M., Sjoholm A., Graubaum H.J., Gruenwald J., Zunft H.J.F., Lueder W. (2007). Cholesterol-lowering effects of a new isolated soy protein with high levels of nondenaturated protein in hypercholesterolemic patients. Adv. Ther..

[140] Ye Y.-B., Tang X.-Y., Verbruggen M.A., Su Y.-X. (2006). Soy isoflavones attenuate bone loss in early postmenopausal Chinese women: A single-blind randomized, placebo-controlled trial. Eur. J. Nutr..

[141] Lethaby A.E., Brown J., Marjoribanks J., Kronenberg F., Roberts H., Eden J. (2007). Phytoestrogens for vasomotor menopausal symptoms. Cochrane Database Syst. Rev..

[142] Farquhar C.M., Marjoribanks J., Lethaby A., Lamberts Q., Suckling J.A., Cochrane HT Study Group (2005). Long term hormone therapy for perimenopausal and postmenopausal women. Cochrane Database Syst. Rev..

[143] Turner R., Baron T., Wolffram S., Minihane A.M., Cassidy A., Rimbach G., Weinberg P.D. (2004). Effect of circulating forms of soy isoflavones on the oxidation of low density lipoprotein. Free Radic. Res..

[144] Lund T.D., Munson D.J., Haldy M.E., Setchell K.D.R., Lephart E.D., Handa R.J. (2004). Equol is a novel anti-androgen that inhibits prostate growth and hormone feedback. Biol. Reprod..

[145] Nagel S.C., vom Saal F.S., Welshons W.V. (1999). Developmental effects of estrogenic chemicals are predicted by an in vitro assay incorporating modification of cell uptake by serum. J. Steroid Biochem. Mol. Biol..

[146] Hilakivi-Clarke L., de Assis S. (2006). Fetal origins of breast cancer. Trends Endocrinol. Metab..

[147] Wang Y., Man Gho W., Chan F.L., Chen S., Leung L.K. (2008). The red clover (Trifolium pratense) isoflavone biochanin A inhibits aromatase activity and expression. Br. J. Nutr..

[148] Vitale D.C., Piazza C., Melilli B., Drago F., Salomone S. (2013). Isoflavones: Estrogenic activity, biological effect and bioavailability. Eur. J. Drug Metab. Pharmacokinet..

[149] Evers N.M., van de Klundert T.M.C., van Aesch Y.M., Wang S., de Roos W.K., Romano A., de Haan L.H.J., Murk A.J., Ederveen A.G.H., Rietjens I.M.C.M. (2013). Human T47D-ERβ breast cancer cells with tetracycline-dependent ERβ expression reflect ERα/ERβ ratios in rat and human breast tissue. Toxicol. In Vitro.

[150] Mueller S.O., Simon S., Chae K., Metzler M., Korach K.S. (2004). Phytoestrogens and their human metabolites show distinct agonistic and antagonistic properties on estrogen receptor α (ERα) and ERβ in human cells. Toxicol. Sci..

[151] Krebs E.E., Ensrud K.E., MacDonald R., Wilt T.J. (2004). Phytoestrogens for treatment of menopausal symptoms: A systematic review. Obstet. Gynecol..

[152] Howes L.G., Howes J.B., Knight D.C. (2006). Isoflavone therapy for menopausal flushes: A systematic review and meta-analysis. Maturitas.

[153] Jou H.-J., Wu S.-C., Chang F.-W., Ling P.-Y., Chu K.S., Wu W.-H. (2008). Effect of intestinal production of equol on menopausal symptoms in women treated with soy isoflavones. Int. J. Gynaecol. Obstet..

[154] Chandrareddy A., Muneyyirci-Delale O., McFarlane S.I., Murad O.M. (2008). Adverse effects of phytoestrogens on reproductive health: A report of three cases. Complement. Ther. Clin. Pract..

[155] Anthony M.S., Clarkson T.B., Hughes C.L., Morgan T.M., Burke G.L. (1996). Soybean isoflavones improve cardiovascular risk factors without affecting the reproductive system of peripubertal rhesus monkeys. J. Nutr..

[156] Zhan S., Ho S.C. (2005). Meta-analysis of the effects of soy protein containing isoflavones on the lipid profile. Am. J. Clin. Nutr..

[157] Sacks F.M., Lichtenstein A., Van Horn L., Harris W., Kris-Etherton P., Winston M., American Heart Association Nutrition Committee (2006). Soy protein, isoflavones, and cardiovascular health: An American Heart Association Science Advisory for professionals from the Nutrition Committee. Circulation.

[158] Reynolds K., Chin A., Lees K.A., Nguyen A., Bujnowski D., He J. (2006). A meta-analysis of the effect of soy protein supplementation on serum lipids. Am. J. Cardiol..

[159] Landmesser U., Hornig B., Drexler H. (2004). Endothelial function: A critical determinant in atherosclerosis?. Circulation.

[160] Mäkelä S., Savolainen H., Aavik E., Myllärniemi M., Strauss L., Taskinen E., Gustafsson J.A., Häyry P. (1999). Differentiation between vasculoprotective and uterotrophic effects of ligands with different binding affinities to estrogen receptors α and β. Proc. Natl. Acad. Sci. USA.

[161] Katz D.L., Evans M.A., Njike V.Y., Hoxley M.L., Nawaz H., Comerford B.P., Sarrel P.M. (2007). Raloxifene, soy phytoestrogens and endothelial function in postmenopausal women. Climacteric.

[162] Evans M., Njike V.Y., Hoxley M., Pearson M., Katz D.L. (2007). Effect of soy isoflavone protein and soy lecithin on endothelial function in healthy postmenopausal women. Menopause.

[163] Teede H.J., Giannopoulos D., Dalais F.S., Hodgson J., McGrath B.P. (2006). Randomised, controlled, cross-over trial of soy protein with isoflavones on blood pressure and arterial function in hypertensive subjects. J. Am. Coll. Nutr..

[164] Liu X.X., Li S.H., Chen J.Z., Sun K., Wang X.J., Wang X.G., Hui R.T. (2012). Effect of soy isoflavones on blood pressure: A meta-analysis of randomized controlled trials. Nutr. Metab. Cardiovasc. Dis..

[165] Levine J.P. (2007). Effective strategies to identify postmenopausal women at risk for osteoporosis. Geriatrics.

[166] Onoe Y., Miyaura C., Ohta H., Nozawa S., Suda T. (1997). Expression of estrogen receptor β in rat bone. Endocrinology.

[167] Canalis E., Giustina A., Bilezikian J.P. (2007). Mechanisms of anabolic therapies for osteoporosis. N. Engl. J. Med..

[168] Cheong J.M.K., Martin B.R., Jackson G.S., Elmore D., McCabe G.P., Nolan J.R., Barnes S., Peacock M., Weaver C.M. (2007). Soy isoflavones do not affect bone resorption in postmenopausal women: A dose-response study using a novel approach with 41Ca. J. Clin. Endocrinol. Metab..

[169] Alekel D.L., Germain A.S., Peterson C.T., Hanson K.B., Stewart J.W., Toda T. (2000). Isoflavone-rich soy protein isolate attenuates bone loss in the lumbar spine of perimenopausal women. Am. J. Clin. Nutr..

[170] Arjmandi B.H., Lucas E.A., Khalil D.A., Devareddy L., Smith B.J., McDonald J., Arquitt A.B., Payton M.E., Mason C. (2005). One year soy protein supplementation has positive effects on bone formation markers but not bone density in postmenopausal women. Nutr. J..

[171] Chen Y.-M., Ho S.C., Lam S.S.H., Ho S.S.S., Woo J.L.F. (2004). Beneficial effect of soy isoflavones on bone mineral content was modified by years since menopause, body weight, and calcium intake: A double-blind, randomized, controlled trial. Menopause.

[172] Huang H.-Y., Yang H.-P., Yang H.-T., Yang T.-C., Shieh M.-J., Huang S.-Y. (2006). One-year soy isoflavone supplementation prevents early postmenopausal bone loss but without a dose-dependent effect. J. Nutr. Biochem..

[173] Ishimi Y. (2010). Dietary equol and bone metabolism in postmenopausal Japanese women and osteoporotic mice. J. Nutr..

[174] Taku K., Melby M.K., Nishi N., Omori T., Kurzer M.S. (2011). Soy isoflavones for osteoporosis: An evidence-based approach. Maturitas.

[175] Tousen Y., Ezaki J., Fujii Y., Ueno T., Nishimuta M., Ishimi Y. (2011). Natural S-equol decreases bone resorption in postmenopausal, non-equol-producing Japanese women: A pilot randomized, placebo-controlled trial. Menopause.

[176] Tousen Y., Ishiwata H., Ishimi Y., Ikegami S. (2015). Equol, a Metabolite of Daidzein, Is More Efficient than Daidzein for Bone Formation in Growing Female Rats. Phytother. Res..

[177] Fujioka M., Uehara M., Wu J., Adlercreutz H., Suzuki K., Kanazawa K., Takeda K., Yamada K., Ishimi Y. (2004). Equol, a metabolite of daidzein, inhibits bone loss in ovariectomized mice. J. Nutr..

[178] Youlden D.R., Cramb S.M., Dunn N.A.M., Muller J.M., Pyke C.M., Baade P.D. (2012). The descriptive epidemiology of female breast cancer: An international comparison of screening, incidence, survival and mortality. Cancer Epidemiol..

[179] Van Erp-Baart M.-A.J., Brants H.A.M., Kiely M., Mulligan A., Turrini A., Sermoneta C., Kilkkinen A., Valsta L.M. (2003). Isoflavone intake in four different European countries: The VENUS approach. Br. J. Nutr..

[180] Messina M., Nagata C., Wu A.H. (2006). Estimated Asian adult soy protein and isoflavone intakes. Nutr. Cancer.

[181] Messina M.J., Wood C.E. (2008). Soy isoflavones, estrogen therapy, and breast cancer risk: Analysis and commentary. Nutr. J..

[182] Shin H.-R., Joubert C., Boniol M., Hery C., Ahn S.H., Won Y.-J., Nishino Y., Sobue T., Chen C.-J., You S.-L. (2010). Recent trends and patterns in breast cancer incidence among Eastern and Southeastern Asian women. Cancer Cause Control.

[183] Bardin A., Boulle N., Lazennec G., Vignon F., Pujol P. (2004). Loss of ERβ expression as a common step in estrogen-dependent tumor progression. Endocr. Relat. Cancer.

[184] Lazennec G., Bresson D., Lucas A., Chauveau C., Vignon F. (2001). ERβ inhibits proliferation and invasion of breast cancer cells. Endocrinology.

[185] Sotoca Covaleda A.M., van den Berg H., Vervoort J., van der Saag P., Ström A., Gustafsson J.-A., Rietjens I., Murk A.J. (2008). Influence of cellular ERα/ERβ ratio on the ERα-agonist induced proliferation of human T47D breast cancer cells. Toxicol. Sci..

[186] Islam M.A., Bekele R., Vanden Berg J.H.J., Kuswanti Y., Thapa O., Soltani S., van Leeuwen F.X.R., Rietjens I.M.C.M., Murk A.J. (2015). Deconjugation of soy isoflavone glucuronides needed for estrogenic activity. Toxicol. In Vitro.

[187] Horn-Ross P.L., John E.M., Canchola A.J., Stewart S.L., Lee M.M. (2003). Phytoestrogen intake and endometrial cancer risk. J. Natl. Cancer Inst..

[188] Xu W.H., Zheng W., Xiang Y.B., Ruan Z.X., Cheng J.R., Dai Q., Gao Y.T., Shu X.O. (2004). Soya food intake and risk of endometrial cancer among Chinese women in Shanghai: Population based case-control study. BMJ.

[189] Murray M.J., Meyer W.R., Lessey B.A., Oi R.H., DeWire R.E., Fritz M.A. (2003). Soy protein isolate with isoflavones does not prevent estradiol-induced endometrial hyperplasia in postmenopausal women: A pilot trial. Menopause.

[190] Messina M.J. (2003). Emerging evidence on the role of soy in reducing prostate cancer risk. Nutr. Rev..

[191] Lund T.D., Blake C., Bu L., Hamaker A.N., Lephart E.D. (2011). Equol an isoflavonoid: Potential for improved prostate health, in vitro and in vivo evidence. Reprod. Biol. Endocrinol..

[192] Adams K.F., Chen C., Newton K.M., Potter J.D., Lampe J.W. (2004). Soy isoflavones do not modulate prostate-specific antigen concentrations in older men in a randomized controlled trial. Cancer Epidemiol. Biomark. Prev..

[193] Fischer L., Mahoney C., Jeffcoat A.R., Koch M.A., Thomas B.E., Valentine J.L., Stinchcombe T., Boan J., Crowell J.A., Zeisel S.H. (2004). Clinical characteristics and pharmacokinetics of purified soy isoflavones: Multiple-dose administration to men with prostate neoplasia. Nutr. Cancer.

[194] Yan L., Spitznagel E.L. (2009). Soy consumption and prostate cancer risk in men: A revisit of a meta-analysis. Am. J. Clin. Nutr..

[195] Chang H.C., Doerge D.R. (2000). Dietary genistein inactivates rat thyroid peroxidase in vivo without an apparent hypothyroid effect. Toxicol. Appl. Pharmacol..

[196] Messina M., Redmond G. (2006). Effects of soy protein and soybean isoflavones on thyroid function in healthy adults and hypothyroid patients: A review of the relevant literature. Thyroid.

[197] Chorazy P.A., Himelhoch S., Hopwood N.J., Greger N.G., Postellon D.C. (1995). Persistent hypothyroidism in an infant receiving a soy formula: Case report and review of the literature. Pediatrics.

[198] Dillingham B.L., McVeigh B.L., Lampe J.W., Duncan A.M. (2007). Soy protein isolates of varied isoflavone content do not influence serum thyroid hormones in healthy young men. Thyroid.

[199] Radović B., Mentrup B., Köhrle J. (2006). Genistein and other soya isoflavones are potent ligands for transthyretin in serum and cerebrospinal fluid. Br. J. Nutr..

[200] Hagen G.A., Solberg L.A. (1974). Brain and cerebrospinal fluid permeability to intravenous thyroid hormones. Endocrinology.

[201] Köhrle J., Fang S.L., Yang Y., Irmscher K., Hesch R.D., Pino S., Alex S., Braverman L.E. (1989). Rapid effects of the flavonoid EMD 21388 on serum thyroid hormone binding and thyrotropin regulation in the rat. Endocrinology.

[202] Hillman G.G., Singh-Gupta V., Hoogstra D.J., Abernathy L., Rakowski J., Yunker C.K., Rothstein S.E., Sarkar F.H., Gadgeel S., Konski A.A. (2013). Differential effect of soy isoflavones in enhancing high intensity radiotherapy and protecting lung tissue in a pre-clinical model of lung carcinoma. Radiother. Oncol..

[203] Moosmann B., Behl C. (1999). The antioxidant neuroprotective effects of estrogens and phenolic compounds are independent from their estrogenic properties. Proc. Natl. Acad. Sci. USA.

[204] Ruiz-Larrea M.B., Mohan A.R., Paganga G., Miller N.J., Bolwell G.P., Rice-Evans C.A. (1997). Antioxidant activity of phytoestrogenic isoflavones. Free Radic. Res..

[205] Amigo-Benavent M., Silván J.M., Moreno F.J., Villamiel M., Del Castillo M.D. (2008). Protein quality, antigenicity, and antioxidant activity of soy-based foodstuffs. J. Agric. Food Chem..

[206] Yoon G.-A., Park S. (2014). Antioxidant action of soy isoflavones on oxidative stress and antioxidant enzyme activities in exercised rats. Nutr. Res. Pract..

[207] Wiseman H., O’Reilly J.D., Adlercreutz H., Mallet A.I., Bowey E.A., Rowland I.R., Sanders T.A. (2000). Isoflavone phytoestrogens consumed in soy decrease F(2)-isoprostane concentrations and increase resistance of low-density lipoprotein to oxidation in humans. Am. J. Clin. Nutr..

[208] Djuric Z., Chen G., Doerge D.R., Heilbrun L.K., Kucuk O. (2001). Effect of soy isoflavone supplementation on markers of oxidative stress in men and women. Cancer Lett..

[209] Monteiro N.E.S., Queirós L.D., Lopes D.B., Pedro A.O., Macedo G.A. (2018). Impact of microbiota on the use and effects of isoflavones in the relief of climacteric symptoms in menopausal women – A review. J. Funct. Foods.

[210] Setchell K.D.R., Zhao X., Shoaf S.E., Ragland K. (2009). The pharmacokinetics of S-(-)equol administered as SE5-OH tablets to healthy postmenopausal women. J. Nutr..

[211] Andersen C., Nielsen T.S., Purup S., Kristensen T., Eriksen J., Søegaard K., Sørensen J., Fretté X.C. (2009). Phyto-oestrogens in herbage and milk from cows grazing white clover, red clover, lucerne or chicory-rich pastures. Animal.

[212] Nielsen T.S., Nørgaard J.V., Purup S., Fretté X.C., Bonefeld-Jørgensen E.C. (2009). Estrogenic activity of bovine milk high or low in equol using immature mouse uterotrophic responses and an estrogen receptor transactivation assay. Cancer Epidemiol..

[213] Antignac J., Cariou R., LeBizec B., André F. (2004). New data regarding phytoestrogens content in bovine milk. Food Chem..

[214] Krajčová A., Schulzová V., Lojza J., Křížová L., Hajšlová J. (2010). Phytoestrogens in bovine plasma and milk—LC-MS/MS analysis. Czech J. Food Sci..

[215] Daems F., Jasselette C., Romnee J.-M., Planchon V., Lognay G., Froidmont É. (2015). Validating the use of an ultra-performance liquid chromatography with tandem mass spectrometry method to quantify equol in cow’s milk. Dairy Sci. Technol..

[216] Kuhnle G.G.C., Dell’Aquila C., Aspinall S.M., Runswick S.A., Mulligan A.A., Bingham S.A. (2008). Phytoestrogen content of foods of animal origin: Dairy products, eggs, meat, fish, and seafood. J. Agric. Food Chem..

[217] Kašparovská J., Dadáková K., Lochman J., Hadrová S., Křížová L., Kašparovský T. (2017). Changes in equol and major soybean isoflavone contents during processing and storage of yogurts made from control or isoflavone-enriched bovine milk determined using LC-MS (TOF) analysis. Food Chem..

[218] Atanassova N., McKinnell C., Fisher J., Sharpe R.M. (2005). Neonatal treatment of rats with diethylstilboestrol (DES) induces stromal-epithelial abnormalities of the vas deferens and cauda epididymis in adulthood following delayed basal cell development. Reproduction.

[219] Franke A.A., Custer L.J. (1996). Daidzein and genistein concentrations in human milk after soy consumption. Clin. Chem..

[220] Balakrishnan B., Thorstensen E.B., Ponnampalam A.P., Mitchell M.D. (2010). Transplacental transfer and biotransformation of genistein in human placenta. Placenta.

[221] Franke A.A., Custer L.J., Wang W., Shi C.Y. (1998). HPLC analysis of isoflavonoids and other phenolic agents from foods and from human fluids. Proc. Soc. Exp. Biol. Med..

[222] Irvine C.H., Shand N., Fitzpatrick M.G., Alexander S.L. (1998). Daily intake and urinary excretion of genistein and daidzein by infants fed soy- or dairy-based infant formulas. Am. J. Clin. Nutr..

[223] Lu L.J., Grady J.J., Marshall M.V., Ramanujam V.M., Anderson K.E. (1995). Altered time course of urinary daidzein and genistein excretion during chronic soya diet in healthy male subjects. Nutr. Cancer.

[224] Olea N., Olea-Serrano F., Lardelli-Claret P., Rivas A., Barba-Navarro A. (1999). Inadvertent exposure to xenoestrogens in children. Toxicol. Ind. Health.

[225] Talsness C., Grote K., Kuriyama S., Presibella K., Sterner-Kock A., Poça K., Chahoud I. (2015). Prenatal Exposure to the Phytoestrogen Daidzein Resulted in Persistent Changes in Ovarian Surface Epithelial Cell Height, Folliculogenesis, and Estrus Phase Length in Adult Sprague-Dawley Rat Offspring. J. Toxicol. Environ. Health Part A.

[226] Degen G.H., Janning P., Diel P., Michna H., Bolt H.M. (2002). Transplacental transfer of the phytoestrogen daidzein in DA/Han rats. Arch. Toxicol..

[227] Dinsdale E.C., Chen J., Ward W.E. (2011). Early life exposure to isoflavones adversely affects reproductive health in first but not second generation female CD-1 mice. J. Nutr..

[228] Jefferson W.N., Patisaul H.B., Williams C.J. (2012). Reproductive consequences of developmental phytoestrogen exposure. Reproduction.

[229] Molzberger A.F., Vollmer G., Hertrampf T., Möller F.J., Kulling S., Diel P. (2012). In utero and postnatal exposure to isoflavones results in a reduced responsivity of the mammary gland towards estradiol. Mol. Nutr. Food Res..

[230] Kaludjerovic J., Chen J., Ward W.E. (2012). Early life exposure to genistein and daidzein disrupts structural development of reproductive organs in female mice. J. Toxicol. Environ. Health Part A.

[231] Greathouse K.L., Bredfeldt T., Everitt J.I., Lin K., Berry T., Kannan K., Mittelstadt M.L., Ho S., Walker C.L. (2012). Environmental estrogens differentially engage the histone methyltransferase EZH2 to increase risk of uterine tumorigenesis. Mol. Cancer Res..

[232] Piotrowska K., Baranowska-Bosiacka I., Marchlewicz M., Gutowska I., Noceń I., Zawiślak M., Chlubek D., Wiszniewska B. (2011). Changes in male reproductive system and mineral metabolism induced by soy isoflavones administered to rats from prenatal life until sexual maturity. Nutrition.

[233] Wang W., Zhang W., Liu J., Sun Y., Li Y., Li H., Xiao S., Shen X. (2013). Metabolomic changes in follicular fluid induced by soy isoflavones administered to rats from weaning until sexual maturity. Toxicol. Appl. Pharmacol..

[234] Fukaya T., Funayama Y., Muakami T., Sugawara J., Yajima A. (1997). Does apoptosis contribute follicular atresia and luteal regression in human ovary?. Horm. Res..

[235] Verdin E., Hirschey M.D., Finley L.W.S., Haigis M.C. (2010). Sirtuin regulation of mitochondria: Energy production, apoptosis, and signaling. Trends Biochem. Sci..

[236] Rajah T.T., Peine K.J., Du N., Serret C.A., Drews N.R. (2012). Physiological concentrations of genistein and 17β-estradiol inhibit MDA-MB-231 breast cancer cell growth by increasing BAX/BCL-2 and reducing pERK1/2. Anticancer Res..

[237] Tang S., Hu J., Meng Q., Dong X., Wang K., Qi Y., Chu C., Zhang X., Hou L. (2013). Daidzein induced apoptosis via down-regulation of Bcl-2/Bax and triggering of the mitochondrial pathway in BGC-823 cells. Cell Biochem. Biophys..

[238] Wang J., Xu J., Wang B., Shu F.R., Chen K., Mi M.T. (2014). Equol promotes rat osteoblast proliferation and differentiation through activating estrogen receptor. Genet. Mol. Res..

[239] Strom B.L., Schinnar R., Ziegler E.E., Barnhart K.T., Sammel M.D., Macones G.A., Stallings V.A., Drulis J.M., Nelson S.E., Hanson S.A. (2001). Exposure to soy-based formula in infancy and endocrinological and reproductive outcomes in young adulthood. JAMA.

[240] Churella H.R., Borschel M.W., Thomas M.R., Breen M., Jacobs J. (1994). Growth and protein status of term infants fed soy protein formulas differing in protein content. J. Am. Coll. Nutr..

[241] Lasekan J.B., Ostrom K.M., Jacobs J.R., Blatter M.M., Ndife L.I., Gooch W.M., Cho S. (1999). Growth of newborn, term infants fed soy formulas for 1 year. Clin. Pediatr..

[242] Gilchrist J.M., Moore M.B., Andres A., Estroff J.A., Badger T.M. (2010). Ultrasonographic patterns of reproductive organs in infants fed soy formula: Comparisons to infants fed breast milk and milk formula. J. Pediatr..

[243] Raman D.R., Williams E.L., Layton A.C., Burns R.T., Easter J.P., Daugherty A.S., Mullen M.D., Sayler G.S. (2004). Estrogen content of dairy and swine wastes. Environ. Sci. Technol..

[244] Hutchins S.R., White M.V., Hudson F.M., Fine D.D. (2007). Analysis of lagoon samples from different concentrated animal feeding operations for estrogens and estrogen conjugates. Environ. Sci. Technol..

[245] Dragomirescu A., Andoni M., Craina M. (2015). Endocrine disrupting compounds in environment—A review. J. Food. Agric. Environ..

[246] Hoerger C.C., Wettstein F.E., Bachmann H.J., Hungerbühler K., Bucheli T.D. (2011). Occurrence and Mass Balance of Isoflavones on an Experimental Grassland Field. Environ. Sci. Technol..

[247] Hoerger C.C., Wettstein F.E., Hungerbühler K., Bucheli T.D. (2009). Occurrence and Origin of Estrogenic Isoflavones in Swiss River Waters. Environ. Sci. Technol..

[248] Kuster M., Azevedo D.A., López de Alda M.J., Aquino Neto F.R., Barceló D. (2009). Analysis of phytoestrogens, progestogens and estrogens in environmental waters from Rio de Janeiro (Brazil). Environ. Int..

